# Compensatory Growth of Scots Pine Seedlings Mitigates Impacts of Multiple Droughts Within and Across Years

**DOI:** 10.3389/fpls.2019.00519

**Published:** 2019-04-24

**Authors:** Hannes Seidel, Michael Matiu, Annette Menzel

**Affiliations:** ^1^Professorship of Ecoclimatology, Department of Ecology and Ecosystem Management, TUM School of Life Sciences Weihenstephan, Technical University of Munich, Freising, Germany; ^2^Institute for Earth Observation, EURAC Research, Bolzano, Italy; ^3^Institute for Advanced Study, Technical University of Munich, Garching bei München, Germany

**Keywords:** provenances, growth timing, plasticity, resilience, assisted migration, *Pinus sylvestris*

## Abstract

Tree seedling resistance to and recovery from abiotic stressors such as drought and warming are crucial for forest regeneration and persistence. Selection of more resilient provenances and their use in forest management programs might alleviate pressures of climate change on forest ecosystems. Scots pine forests in particular have suffered frequent drought-induced mortality, suggesting high vulnerability to extreme events. Here, we conducted an experiment using potted Scots pine seedlings from ten provenances of its south-western distribution range to investigate provenance-specific impacts of multiple drought events. Seedlings were grown under ambient and elevated temperatures for 1.5 years and were subjected to consecutive droughts during spring and summer. Growth (height, diameter, and needle) and spring phenology were monitored during the whole study period and complemented by biomass assessments (bud, needle, wood, and needle/wood ratio) as well as measurements of chlorophyll fluorescence and of needle stable carbon isotope ratio. Phenology, growth and biomass parameters as well as carbon isotope ratio and their (direct) responses to reoccurring droughts differed between provenances, indicating genotypic adaptation. Seedling growth was plastic during drought with intra- and inter-annual compensatory growth after drought stress release (carryover effects), however, not fully compensating the initial impact. For (smaller) seedlings from southern/drier origins, sometimes greater drought resistance was observed which diminished under warmer conditions in the greenhouse. Warming increased diameter growth and advanced phenological development, which was (partly) delayed by drought in 2013, but advanced in 2014. Earlier phenology was linked to higher growth in 2013, but interestingly later phenology had positive effects on wood and needle biomass when subjected to drought. Lastly, stable carbon isotope ratios indicated a clear drought response of carbon assimilation. Drought-induced reduction of the photosystem II efficiency was only observed under warmer conditions but showed compensation under ambient temperatures. Besides these direct drought impacts, also interactive effects of previous drought events were shown, either reinforcing or sometimes attenuating the actual impact. Thus, depending on amount and timing of events, Scots pine seedlings, particularly from southern origins, might be well adapted and resilient to drought stress and should be considered when discussing assisted migration under changing climatic conditions.

## Introduction

Responses of temperate tree species to water shortage and rising temperatures are manifold, comprising molecular, physiological, and structural responses (Chaves et al., 2003; Niinemets, 2010). On the molecular level, gene expression pathways of molecules that maintain cell turgor and integrity such as abscisic acid, proline, soluble sugars, heat shock proteins or anti-stress proteins are stimulated (Peñuelas et al., 2013). On the physiological level, drought can reduce photosynthetic activity, stomatal conductance, transpiration, sap flow and carbon assimilation, while increasing water use efficiency (Tognetti et al., 1997; Rennenberg et al., 2006; de Miguel et al., 2012; Arend et al., 2013; Klein et al., 2013). Notably, slight to moderate warming has been observed to trigger the opposite response, e.g., an increase in net photosynthesis, stomatal conductance and specific hydraulic conductivity, and a decrease in water use efficiency (Maherali and DeLucia, 2000; Arend et al., 2013; Zhao et al., 2013). Drought-induced structural responses include leaf shedding, reductions of leaf size, changes in xylem conduit size, declines in leaf/sapwood area ratio, and reductions in total growth along with changes in resource allocation from shoots to roots (Irvine et al., 1998; Peña-Rojas et al., 2005; Eilmann et al., 2009; Martínez-Vilalta et al., 2009; Bryukhanova and Fonti, 2013; Kuster et al., 2013; Taeger et al., 2013a, 2015).

Temperature-growth relationships, on the other hand, are not always uniform: dendroecological studies on Scots pine and Norway spruce have found negative correlations between ring width increment and temperature during the growing season (Pichler and Oberhuber, 2007; Zang et al., 2012), while experimental studies have revealed negative, positive or no effects of temperature on above-ground growth (Olszyk et al., 1998; Arend et al., 2011; Wu et al., 2011; Taeger et al., 2015). Therefore, Saxe et al. (2001) have suggested that temperature responses could be species- or even provenance-specific. Nevertheless, common growth responses to rising temperatures include increasing leaf area and xylem conduits, and decreasing root/shoot ratios and leaf/sapwood area ratios (Maherali and DeLucia, 2000; Way and Oren, 2010).

With future warming along with higher severity and frequency of droughts (Kirtman et al., 2013), climatic pressures are predicted to intensify. Anatomical and physiological changes induced by warmer growing seasons may render trees more susceptible to drought, as reviewed by Way (2013). Since vapor pressure deficit is positively correlated with temperature, warming would furthermore increase the evaporative demand of the atmosphere and thus increase transpiration (Allen et al., 1998; Cinnirella et al., 2002).

The timing of water availability plays a major role for tree growth since foliage, height and diameter show seasonal growth patterns in temperate regions (Anderegg et al., 2013). For example, radial growth of deciduous trees may be most sensitive to early season water deficit whereas that of conifers may also be influenced by late season water deficit (Hanson and Weltzin, 2000). Species that flush early may finish shoot and leaf expansion before drought occurs, while species that exhibit continuous growth and multiple flushes are more strongly affected by seasonal drought events (Bréda et al., 2006). Additionally, past climatic events can have long-lasting effects on tree growth. For instance, current year diameter growth has been shown to be positively correlated with water availability of the previous year (Eilmann et al., 2009; Michelot et al., 2012). Similarly, shoot elongation, leaf number and size depend on the climatic conditions of bud formation in the previous year (Bréda et al., 2006). Furthermore, drought may delay or advance leaf phenology in the following year (Bernal et al., 2011; Misson et al., 2011; Peñuelas et al., 2012; Spieß et al., 2012; Swidrak et al., 2013; Vander Mijnsbrugge et al., 2016). Thus, drought events can have long-lasting impacts on radial, twig and leaf growth that may take several years until pre-drought growth conditions are fully regenerated (Becker, 1989; Orwig and Abrams, 1997; Stribley and Ashmore, 2002; Bréda et al., 2006).

However, compensatory responses following a drought event within and across years are also reported, such as increased radial and shoot growth, additional flushing, earlier leaf development, delayed leaf senescence, and increased cell enlargement rates (Spieß et al., 2012; Taeger et al., 2013b; Balducci et al., 2016; Turcsán et al., 2016; Vander Mijnsbrugge et al., 2016). Scots pine for instance, has the ability to adjust leaf/sapwood area ratio, leaf-specific hydraulic conductivity, total leaf area and conduit size in response to drought (Sterck et al., 2008; Martínez-Vilalta et al., 2009). Additionally, acclimation facilitates enhanced resistance to re-occurring stress events (Seidel and Menzel, 2016).

Scots Pine (*Pinus sylvestris* L.) has a wide-ranging distribution from Spain to Scandinavia to the far east of Russia, covering a number of climatically contrasting environments (boreal to Mediterranean) (Boratynski, 1991). Its general response to water shortage is a drought avoidance strategy characterized by strong stomatal control reducing water loss through the needles (Irvine et al., 1998). This strategy implies reduced photosynthetic carbon gain (Mitchell et al., 2013) and renders Scots pine particularly susceptible to long drought events, even if these events are not severe (Anderegg et al., 2013). Overall, drought has negative effects on Scots pine shoot elongation and radial increment (Irvine et al., 1998; Taeger et al., 2013b). Colonization of fundamentally different climatic regions has favored local adaptations of Scots pine provenances (Boratynski, 1991; Reich and Oleksyn, 2008), resulting in different responses to drought regarding seedling establishment, mortality, shoot elongation and radial increment, stomatal conductance, and drought resistance (Richter et al., 2012; Taeger et al., 2013a,b, 2015; Seidel and Menzel, 2016; Seidel et al., 2016). Although Scots pine is considered to be a drought resistant species (Ellenberg, 1988), numerous drought-induced mortality events of Scots pine forests have been documented in the last decades (Allen et al., 2010). Recovery from disturbances as well as forest regeneration and persistence might depend on seedling vitality and, furthermore, be crucial to alleviate climate change impacts. Thus, forest management might consider assisted migration of suitable provenances adapted to warmer and/or drier climates of the species’ distribution range (Millar et al., 2007; Bussotti et al., 2015).

Climate-growth relationships of Scots pine have been frequently investigated (Oberhuber et al., 1998; Linderholm, 2001; Rigling et al., 2002; Pichler and Oberhuber, 2007; Martínez-Vilalta et al., 2008; Michelot et al., 2012; Sánchez-Salguero et al., 2012, 2015; Zang et al., 2012), while provenance-specific drought impacts on growth are less studied (Cregg and Zhang, 2001; Richter et al., 2012; Taeger et al., 2013a,b, 2015). Knowledge about the influence of seasonal drought events on provenance-specific intra- and inter-annual growth and ecophysiological responses of Scots pine seedlings is lacking in particular.

We therefore ask the following research questions: (1a) How do Scots pine seedlings respond to drought (direct drought effects), (1b) do provenances differ in their drought response, and (1c) are those differences linked to the climate at their origin assuming local adaptation? (2) Are drought impacts additive or can Scots pine seedlings acclimate to drought, e.g., through growth reductions, in order to increase resistance to subsequent drought events within and across years (interactive drought effects)? (3) After drought stress release, do negative effects of drought persist or do Scots pine seedlings recover and even compensate negative effects (carryover drought effects)? In this study, we conducted a seasonal drought experiment using potted seedlings of 10 European Scots pine provenances to evaluate the intra- and inter-annual impact on growth and ecophysiological response across one and a half years. Drought conditions were simulated in the spring and summer of the 1st year and in the spring of the 2nd year. Drought periods were intermitted by well-watered conditions. Additionally, seedlings were grown under ambient temperature and under passively elevated temperatures in a greenhouse.

## Materials and Methods

### Plant Material and Growing Conditions

We used Scots pine seedlings of 10 provenances originating from their south-western distribution, namely from Poland (PL9: Suprasl), Germany (D8: Mittel-/Ostdt. Tiefland, D6: Hauptsmoorwald, D7: Alpenkiefer), Hungary (HU14: Plantage Pornoapati), Italy (I4: Emilia Romagna), France (F12: Mont Ventoux, F3: Prealpes du Sud), Spain (ES1: Alto Ebro) and Bulgaria (BG10: Garmen). Hereafter, provenances are referred using their abbreviations, which combine the international vehicle registration or country code (ISO 3166-1 alpha-2) and an internal number (Table 1). An isozyme study of Taeger et al. (2013a) revealed that German provenances, PL9 and BG10 belong to the same gene pool, whereas I4, F12, F3, and ES1 each belong to a separate gene pool. Annual mean temperature and sum of precipitation at the origin of seed sources ranged between ∼3 and 11°C and ∼600 and 1,500 mm, respectively (Table 1). Aside from D7 and HU14, which were from seed orchards, seeds were collected from autochthonous populations. In April 2012, 232–241 one-year-old seedlings per provenance were potted in 3 l pots containing a peat substrate (‘Basismischung Bayer. Staatsforsten AöR’, Klasmann-Deilmann GmbH, Geeste, Germany) at the Gewächshauslaborzentrum (GHL) near Freising, Germany. Half of the plants per provenance were placed in a vegetation hall (a glassed roofed building with open side walls), and the second half of seedlings was arranged in a greenhouse, creating two different temperature regimes: close to ambient conditions in the vegetation hall and a 3°C passive warming in the greenhouse [see Seidel and Menzel (2016) for detailed information]. Hereafter, if not specified otherwise, statements apply to the greenhouse as well as to the vegetation hall. The seedlings were randomly arranged on three tables within each building, with similar numbers of individuals per provenance on each table. Plants were acclimated to the local growing conditions and, based on biweekly soil moisture measurements, they were kept well-watered using a time-controlled dripping system until the experimental drought manipulations were conducted from March 2013 to June 2014. Due to partial harvest of Scots pine seedlings in November 2013 and the use of individuals from specific provenances for another experiment (Lüpke et al., 2016), only around 80 individuals from seven provenances each remained available in 2014 to be distributed across the two buildings and watering regimes of 2013. In March 2014, these remaining seedlings were replanted into 20 l pots containing substrate of identical composition as before and were again randomly arranged on tables within each building, with similar numbers of individuals per provenance and watering regime on each table. For further analyses of the experiment conducted in 2014, these seedlings were grouped according to their latitude into northern (>45°N) and southern (<45°N) provenances (Table 1), termed region in the subsequent manuscript. The climate at the origin of northern provenances shows a distinct precipitation maximum in summer whereas there is a distinct precipitation minimum at the origin of southern provenances (Supplementary Figure S1).

**Table 1:** Origin of seed sources.

Provenance abbreviation	Provenance	Country	Region (north: >45°N; south: <45°N)	Lat.	Long.	Alt. (m a.s.l.)	T (°C)	PPT (mm)	AI _*annual*_	AI _*growingseason*_	AI _*summer*_	AI _*minimum*_	N 2013	N 2014
PL9	Suprasl	Poland	North	53°15′N	23°23′E	181	7.00	569	0.94	0.75	0.67	0.56	238	80
D8	Mittel-/Ostdt. Tiefland	Germany	North	53°04′N	13°29′E	75	8.66	594	0.90	0.68	0.59	0.53	240	0
D6	Hauptsmoorwald	Germany	North	49°51′N	10°58′E	250	8.57	609	0.95	0.75	0.67	0.62	237	80
D7	Alpenkiefer	Germany	North	47°30′N	11°20′E	1150	3.24	1517	2.73	2.22	2.05	1.82	241	80
HU14	Plantage Pornoapati	Hungary	North	47°20′N	16°28′E	300	9.04	793	1.10	0.93	0.89	0.77	232	80
I4	Emilia Romagna	Italy	South	44°30′N	10°27′E	460	8.27	942	1.30	0.96	0.56	0.41	238	0
F12	Mont Ventoux	France	South	44°10′N	05°16′E	1600	10.30	984	1.05	0.81	0.48	0.35	241	79
F3	Prealpes du Sud	France	South	43°45′N	06°40′E	1185	11.41	947	0.99	0.75	0.42	0.29	238	79
ES1	Alto Ebro	Spain	South	42°59′N	03°17′W	860	10.00	851	1.03	0.72	0.42	0.33	239	0
BG10	Garmen	Bulgaria	South	41°43′N	23°54′E	1300	8.78	653	0.70	0.55	0.41	0.32	240	80

*Geographic location, altitude, annual mean temperature (T), annual sum of precipitation (PPT). Annual, growing season (March–October), summer (June–August) and monthly minimum aridity index (AI) defined as ratio of precipitation to potential evapotranspiration as well as number of replicates (N) of seed sources. T, PPT and AI are shown as means for the period 1950–2000 (CRU TS 4.02, Harris et al., 2014). Months of minimum AI were May for PL9–HU14, July for I4–ES1 and August for BG10. The climate at the origin of northern provenances shows a distinct precipitation maximum in summer, whereas there is a distinct precipitation minimum at the origin of southern provenances (Supplementary Figure S1)*.

### Drought Treatments

**Figure 1:**
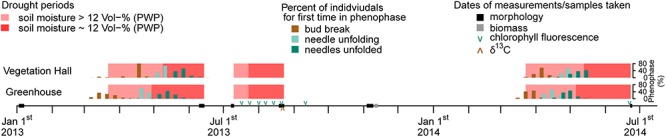
Timeline for the experimental period from January 1, 2013 till July 1, 2014 with seasonal drought periods, phenophases and dates of measurements and sample collection. Note that some individuals in 2014 did not unfold all needles till June 3, 2014 and were therefore not observed furthermore. Bars of the percentages of phenophases are slightly shifted to avoid hiding each other.

Drought conditions were simulated during a spring and a summer period in 2013 and a spring period in 2014 (Figure 1). In between all seedlings were well-watered (recovery periods). The design of the time-controlled dripping system enabled a maximum of four different seasonal watering groups per year.

In March 2013, 28–31 seedlings per provenance and building were assigned to a well-watered control treatment (wet–wet), a spring drought treatment (March 22 to June 14, dry–wet), a summer drought treatment (July 10 to August 21, wet–dry) or a spring and summer drought treatment (dry–dry). Hereafter, seasonal drought treatments are referred to in shortened form (i.e., spring drought or summer drought). During the drought treatments soil moisture was adjusted to oscillate around the permanent wilting point (pF 4.2) through an initial dry-off period and a subsequent addition of small amounts of water when necessary (Supplementary Figure S2). The permanent wilting point was derived from water retention curves following the pressure plate method described by Richards (1941) and corresponded to 12 Vol% soil moisture, which was achieved during spring for around 5 weeks in the vegetation hall and 6 weeks in the greenhouse, and during summer for around 4 weeks in both buildings (Figure 1 and Supplementary Figure S2). All seedlings received equal amounts of water after the drought treatments, which were considered to be a recovery period until the next drought treatment began.

In 2014, 28–40 individuals per building, regional provenance group (north and south) and drought treatment group of 2013 (wet–wet, wet–dry, dry–wet, and dry–dry) were exposed to a drought treatment from March 23 to June 23 by totally withholding irrigation. The soil moisture fell below the permanent wilting point for around 5 weeks in the vegetation hall and 6 eeks in the greenhouse (Figure 1 and Supplementary Figure S2).

Soil moisture was monitored twice a week using a hand-held soil moisture sensor (UMP1, Umwelt-Geräte-Technik GmbH, Müncheberg, Germany) on 240 pots equally spread across provenances and treatments (Supplementary Figure S2). Due to technical problems with the dripping system during summer 2014, we did not analyze the recovery period after the 2014 drought treatment.

### Phenological, Morphological, and Ecophysiological Measurements

#### Phenology

Phenology was monitored weekly on the terminal buds of the main shoot of all individuals from March 3 to June 28, 2013, and from March 17 to June 2, 2014 (Figure 1). We recorded the onset of three different phenophases which were classified as bud break (first green tissue visible between bud scales), needle unfolding (first needles emerging from needle sheaths) and needles unfolded (all needles have penetrated through needle sheaths) (Hack et al., 1992).

#### Height, Diameter, Needle Length and Above-Ground Dry Weight

Morphology of seedlings was monitored during five periods in 2013 and one period in 2014; namely from January 3 to 7, 2013 (before the start of the growing season), from June 10 to 14, 2013 (at the end of the spring drought period), from July 8 to 10 and from August 19 to 23, 2013 (before and at the end of the summer drought period), in autumn from November 4 to 9 in 2013, and on June 23 and 24, 2014 following the spring drought period (Figure 1). Height was measured from the substrate surface to the terminal tip of each plant using a digital caliper in 2013 and a folding ruler in 2014. Diameter was determined using a digital caliper at the height of the pot rim, while needle length was measured using a customized ruler at 2 mm intervals. Mean needle length per individual was determined using ten needles equally distributed along the terminal shoot of the respective year. Height and diameter were assessed on all 2,384 pine individuals whereas mean needle length was evaluated for twelve individuals per building, provenance and drought treatment group. From November 11 to 13, 2013, we harvested 20 seedlings per building, provenance and drought treatment group, to assess the dry weight of above-ground compartments. Seedlings were thus cut at the root collar, oven dried at 60°C for 48 h and then separated into wood, needles and buds.

#### Quantum Efficiency of Photosystem II

Quantum efficiency of photosystem II (PSII) was used as a stress indicator of the photochemical efficiency since it is sensitive to drought conditions (Maxwell and Johnson, 2000; Ashraf and Harris, 2013). Quantum efficiency of PSII was measured on one dark-adapted needle pair of the current year shoot per individual with a continuous excitation fluorimeter (Pocket PEA, Hansatech Instruments Ltd., Pentney, United Kingdom) from midnight until 4 am. We took measurements on nine individuals per building, provenance and drought treatment group in 2013 (720 individuals in total) and on all individuals in 2014 (560 individuals in total). Sampling was conducted on identical individuals in eight periods, six times during the summer drought in 2013, once 3 weeks after the summer drought in 2013 (recovery) and once at the end of the spring drought in 2014 (Figure 1). Due to the large amount of individuals, each sampling period consisted of two consecutive nights during which equal numbers of randomly chosen individuals per building, provenance and drought treatment group were measured.

#### Stable Carbon Isotope Ratio of Needles

We selected the four provenances D8, I4, ES1, and BG10 that had a low genetic relationship and differed in their drought response (Taeger et al., 2013a). Needle samples were taken at the end of the summer drought period in 2013 (Figure 1). We collected ten needle pairs from the current year main shoot of 10 random individuals per building, drought treatment group and provenance, resulting in 320 samples. Needles were oven dried at 60°C for 48 h, grounded with liquid nitrogen and weighed before using an elemental analyzer (Euro EA 3000, Eurovector S.p.A, Milan, Italy) and mass spectrometer (Isoprime, GV Instruments Ltd., Manchester, United Kingdom) for isotopic analyses.

### Statistical Analyses

Linear models using generalized least squares (nlme; Pinheiro et al., 2015) implemented in the R version 3.3.2 (R Core Team, 2016) were applied separately to the vegetation hall and the greenhouse to analyze the effects of provenance, region, and drought on (1) phenology, (2) height, diameter and needle growth, (3) wood, needle, bud and total above ground biomass as well as the needle to wood ratio, (4) quantum efficiency of PSII after the summer drought in 2013 and during the drought in 2014, (5) stable carbon isotope ratio, and (6) to analyze the association between structural response (growth, biomass) and physiological response (stable carbon isotope ratio). For the analysis of the quantum efficiency of PSII during the summer drought in 2013 we used linear mixed effects models (nlme, Pinheiro et al., 2015), with the individual seedlings represented as random variables to account for repeated measurements.

Intra-annual growth of 2013 was analyzed for multiple periods until the growth of respective compartments was completed (Supplementary Figure S3); these included January to June and June to July for height growth, January–June, June–July, July–August, and August–November for diameter growth, and January–June, June–July, and July–August for needle growth. Additionally, we analyzed the annual growth of 2013 and growth from November 2013 to June 2014.

The most complex statistical models (full models) included all possible explanatory terms of provenance or region and drought treatments as factorial dummy variables. Only those drought treatments were included in the full models which occurred before and during the respective time or period of measurement of the dependent variable. We included the additional covariates phenophase and/or morphological measures at the beginning of the experiment to evaluate the provenance-specific drought response of height growth, diameter growth and biomass independent of growth potential and growth timing. Therefore, models of height growth included the covariates height that was measured in January along with bud break, i.e., the start of shoot elongation. Diameter growth models included bud break and needle unfolding apart from the diameter measured in January, as the beginning of radial growth can be related to the start of shoot or needle growth depending on environmental conditions (Swidrak et al., 2013). Needle growth models contained the phenophase of needle unfolding, and biomass models used bud break and needle unfolding as additional covariates. All possible high-order interactions of categorical dummy variables and all possible interactions of current year drought treatments with continuous covariates were included in the statistical full models except for needle growth to prevent overfitting because of a smaller sample size.

Models evaluating the association between structural and physiological response included drought treatments and their interaction as well as provenance, stable carbon isotope ratio and their interaction.

Diagnostic plots were checked for heteroscedasticity and non-normal distribution of the residuals; variance function structure classes were applied whenever needed. The full models were then simplified based on minimizing the AIC using the drop1 function (stats; R Core Team, 2016). Furthermore, the significance of single terms was proven by performing a type III ANOVA using the Anova function (car; Fox and Weisberg, 2011). These test statistics can be found in Supplementary Tables S1–S14. Pairwise comparisons of treatments, provenances, regions and their interactions were computed with the lsmeans function adjusting *p*-values with the “fdr” method (Lenth, 2016).

If the drought impact was still detectable after drought stress release, we considered it a carry-over drought effect. If a previous drought altered the resistance to a subsequent drought event either through acclimation or additive impacts, we called it interactive drought effect.

When analyses of growth parameters revealed a differing drought response for provenances or regional groups, we calculated their absolute and relative response magnitude. Particular response magnitudes were calculated by pairwise subtracting each individual’s growth of the control group from each individual’s growth of the drought group. Analyses of response magnitudes were done using generalized least squares (nlme; Pinheiro et al., 2015) with provenance or region as explanatory variable and the lsmeans function (Lenth, 2016) for pairwise comparisons adjusting *p*-values with the “fdr” method.

Adaptation of provenances’ mean annual growth, mean biomass and the aforementioned mean drought response to local site aridity [aridity index (AI) = ratio of precipitation to potential evapotranspiration) was analyzed by conducting simple linear regressions with annual AI, growing season AI, summer AI and the minimum AI of the year using the lm function (stats; R Core Team, 2016). D7 was excluded from this study because AI values exceed the AI values of the second moist site by more than 100% (Table 1).

## Results

### Influence of Provenance and Drought on Phenology 2013

Mean bud break, needle unfolding and needles unfolded in 2013 occurred 25, 17, and 11 days earlier in the warmer greenhouse than in the vegetation hall, respectively (Supplementary Figure S4) and mostly varied with provenance (except needle unfolding in the greenhouse; Supplementary Table S1). Except for bud break in the greenhouse, the onset of all phenophases was earliest for PL9 (Supplementary Figure S4), e.g., in the vegetation hall mean PL9 bud break was almost 6 days earlier than F12 (*p* = 0.001) and mean needle unfolding 5 days earlier than ES1. Bud break in general and needle unfolding in the greenhouse occurred before the start of the spring treatment 2013 (Figure 1) and were consequently not affected by the drought. The spring drought then delayed needle unfolding by 1.3 days (*p* < 0.001; Supplementary Table S1 and Supplementary Figure S4B) exclusively in the vegetation hall and after that more strongly the later phenological phase of needles unfolded in both buildings by more than 5 days (see also Figure 4A).

### Annual Variations of Seedling Growth and Wood, Needle and Bud Biomass in 2013

**Figure 2:**
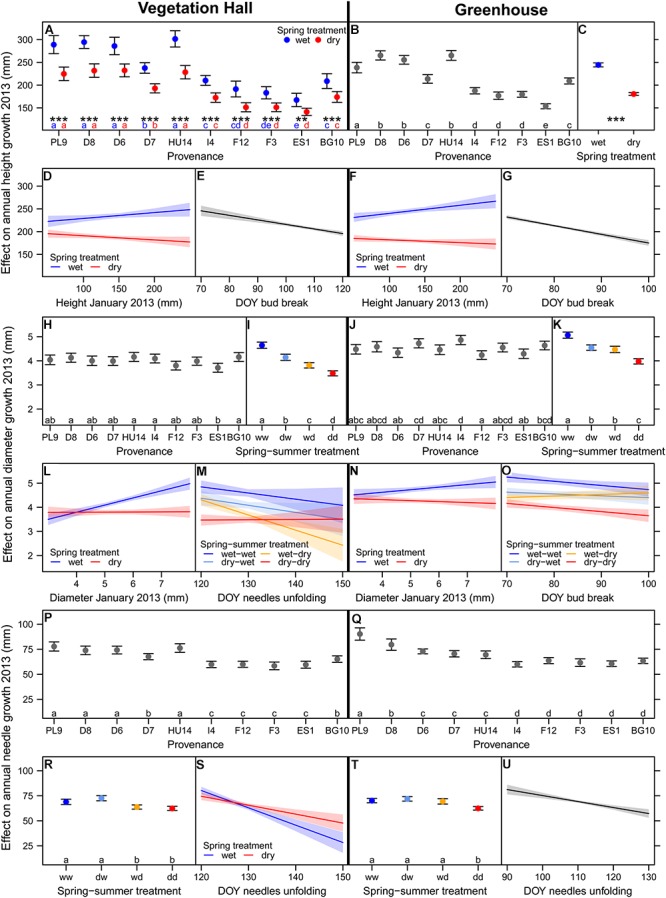
Estimated model effects of explanatory variables included in the final model on **(A–G)** annual height growth, **(H–O)** annual diameter growth, and **(P–U)** annual needle growth in 2013 in **(A,D,E,H,I,L,M,P,R,S)** the vegetation hall and in **(B,C,F,G,J,K,N,O,Q,T,U)** the greenhouse. Shown are fitted mean values and 95% confidence intervals for each variable holding all other variables constant around their mean. Abbreviations in **(I,K,R,T)** denote wet (w) and dry (d) conditions during the spring and summer treatment. Provenances and treatments sharing the same lowercase letter in the same color within buildings are not different at a significance level of 0.05. Significance levels of asterisk are ^∗∗∗^*p* < 0.001, ^∗∗^*p* < 0.01.

Mean annual height and needle growth was comparable in the vegetation hall and in the greenhouse, whereas overall mean annual diameter growth was 0.5 mm higher in the greenhouse than in the vegetation hall (Figure 2). Height, diameter and needle growth varied with provenance (Supplementary Tables S2–S4). The southern provenances generally had lower height and needle growth (Figure 2A,B,P,Q; at least *p* < 0.02). In the vegetation hall, ES1 had the smallest diameter growth and differed significantly (*p* < 0.05) from D8, I4 and BG10 which had the highest diameter growth (Figure 2H). In the greenhouse, I4 had the highest diameter growth (Figure 2J) and significantly exceeded (at least *p* < 0.04) the growth of PL9, D6, HU14, F12, and ES1. All measured biomass parameters significantly varied with provenance (Supplementary Table S5), but did not show a clear regional pattern and thus are not presented in more detail. In some cases, the provenances from seed orchards (D7 and HU14) are among the better performing provenances (Supplementary Figure S5).

**Figure 3:**
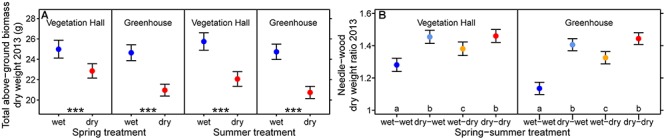
Estimated model effects of the spring and the summer treatment on **(A)** total above-ground biomass dry weight and **(B)** needle to wood dry weight ratio in 2013. Shown are fitted mean values and 95% confidence intervals holding all other variables included in the final model constant around their mean. Significance level of asterisk are ^∗∗∗^*p* < 0.001. Treatments sharing the same lowercase letter within buildings are not different at a significance level of 0.05.

The direct negative effect of spring drought on mean height growth was larger in the warmer greenhouse (-64 mm, -26%) than in the vegetation hall (-46 mm, -20%), although in the vegetation hall this effect depended on the provenance, i.e., northern provenances had a higher absolute reduction than southern ones (at least *p* < 0.002; Figure 2A,C and Supplementary Figure S6A), but still grew taller than southern provenances. Spring and summer drought both separately significantly reduced total above-ground biomass (*p* < 0.001, Figure 3A and Supplementary Table S5) and dry weight of above-ground wood and buds (Supplementary Figures S7A,C and Supplementary Table S5).

Interacting effects of spring and summer drought were observed for annual diameter growth, needle growth, needle biomass and the needle to wood ratio (Figure 2I,K,R,T and Supplementary Tables S3–S5). In the vegetation hall, we observed a stepwise decline in diameter growth from spring (-0.5 mm, -11%) to summer drought (-0.8 mm, -18%) to spring and summer drought (-1.2 mm, -25%), whereas in the greenhouse, spring and summer drought caused a similar decrease (*p* < 0.001) by around -0.5 mm (-10%) in contrast to -1.1 mm (-21%) for both drought treatments (*p* < 0.001; Figure 2I,K). Needle growth decreased under summer drought conditions in the vegetation hall (Figure 2R) while under greenhouse conditions only the combination of both droughts had a negative effect (*p* < 0.001; Figure 2T). The pattern of annual needle growth was also reflected in needle dry weight (Supplementary Figure S7B and Supplementary Table S5). The needle to wood ratio of seedlings increased with drought regardless of season, but less strongly when only subjected to a summer drought (at least *p* < 0.002; Figure 3B and Supplementary Table S5).

Initial height and diameter in January 2013 promoted respective annual growth when seedlings got well-watered in spring, but had no effect on annual diameter growth or even slightly decreased annual height growth under spring drought conditions (Figure 2D,F,L,N and Supplementary Tables S2, S3). Later bud break resulted in lower annual height growth (Figure 2E,G and Supplementary Table S2). This effect was stronger in the greenhouse (-1.9 mm/day) than in the vegetation hall (-1.0 mm/day). Later bud break decreased annual diameter growth, except for the summer drought group, and increased the needle to wood ratio just under warmer conditions in the greenhouse (Figure 2O and Supplementary Tables S3, S5). Needle unfolding interacting with spring and summer drought improved the model fit of diameter growth in the vegetation hall, although the slopes of the treatment groups were not significantly different from the well-watered control group (Figure 2M). Later needle unfolding generally resulted in shorter needles, with a weaker response under spring drought conditions (Figure 2S,U and Supplementary Table S4) and increased bud weight in the greenhouse (Supplementary Table S5). A later start of needle unfolding had positive effects on wood and needle biomass when subjected to summer drought and combined spring and summer drought conditions, respectively (Supplementary Table S5). Additionally, later needle unfolding decreased the needle to wood ratio in the spring drought treatment (greenhouse) and summer drought treatment (vegetation hall) (Supplementary Table S5).

### Intra-Annual Variations of Seedling Growth in 2013 With Provenance, Morphology and Phenology

Height growth from January to June and June to July as well as needle growth from January to June, June to July, and July to August clustered provenances according to their origin (north and south, Supplementary Figures S8A,B,E–G). In general, southern provenances grew less and were less affected during drought periods in terms of absolute height growth in the vegetation hall as well as absolute needle growth from July to August in the greenhouse (Supplementary Figures S6A,B). In contrast, intra-annual diameter growth was not associated with a north–south pattern (Supplementary Figures S8C,D).

During the growth period from January to June, taller seedlings grew more under well-watered conditions but were more strongly affected by drought conditions. A later bud break generally resulted in smaller seedlings (Supplementary Table S2).

The initial diameter measured in January increased intra-annual diameter growth under well-watered conditions, but decreased it under drought. In the vegetation hall, a later start of needle unfolding resulted in less intra-annual diameter growth from January to June (only in the control) and from August to November, but promoted diameter growth during the period from June to July. In the greenhouse, seedlings that started to unfold needles later had smaller diameter growth from July to August (Supplementary Table S3).

Except during July and August, later start of needle unfolding resulted in shorter needles in the greenhouse. Needle growth of seedlings responded less during drought in the vegetation hall, and when released from drought from June to July in the greenhouse (Supplementary Table S4).

### Direct, Interactive and Carryover Drought Effects on Intra-Annual Growth in 2013

**Figure 4:**
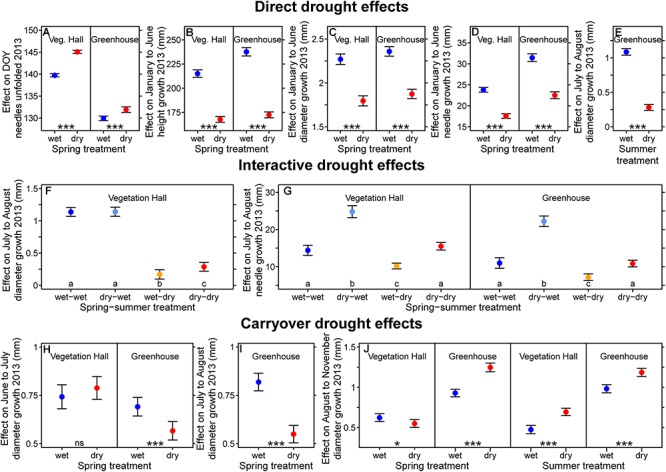
Estimated **(A–E)** direct, **(F,G)** interactive, and **(H–J)** carryover drought model effects on **(A)** the day when needles were unfolded, **(B)** intra-annual height growth, **(C,E,F,H–J)** intra-annual diameter growth and **(D,G)** intra-annual needle growth in 2013. **(F,G)** Drought resilient growth patterns were observed for diameter growth. **(J)** Diameter and **(G)** needle growth show compensatory growth after drought stress release. Shown are fitted mean values and 95% confidence intervals holding all other variables included in the final model constant around their mean. Provenances and treatments sharing the same lowercase letter within the vegetation hall (Veg. Hall) and the greenhouse are not different at a significance level of 0.05. Levels of significance are ^∗^*p* < 0.05, ^∗∗^*p* < 0.01, ^∗∗∗^*p* < 0.001; and ns *p* > 0.05.

During spring, drought significantly reduced height, diameter and needle growth, and delayed needles unfolded in the vegetation hall and the greenhouse by 5.2 and 2.0 days, respectively (*p* < 0.001; Figure 4A–D). Although the absolute growth was higher in the greenhouse, the relative reduction was similar in both buildings and ranged from 22 to 28%. During drought in summer, diameter growth even decreased by 75% in the greenhouse (*p* < 0.001; Figure 4E).

In the vegetation hall, the reduction of diameter growth during the summer treatment period was influenced by previous spring treatment conditions (Figure 4F). These interactive drought effects (dependency of the effect of a drought on a previous drought) were detected not only for July to August diameter growth, but also needle growth (Supplementary Tables S3, S4 and Figure 4G). In the vegetation hall, diameter growth from July to August was significantly lower (*p* < 0.001) in individuals experiencing the summer drought, but seedlings that additionally experienced the spring drought before being subjected to the summer drought had 70% higher (!) diameter growth (*p* = 0.024) than seedlings that only experienced the summer drought (Figure 4F). From July to August in both buildings, the summer drought alone decreased needle growth by around 30% compared to the control (at least *p* < 0.008), while seedlings that experienced the spring and summer drought showed resistance and grew similarly to control seedlings (Figure 4G). Seedlings that experienced drought during spring exhibited a 72% (vegetation hall) and 102% (greenhouse) increase of July to August needle growth (*p* < 0.001; Figure 4G) compared to the control.

Diameter growth of pine seedlings was influenced by drought periods following the release from respective drought stress (carryover drought effects; Figure 4H–J). In the greenhouse, the negative effect of the spring drought persisted for diameter growth from June to July and July to August (*p* < 0.001; Figure 4H,I). In the spring drought group, diameter growth from August to November was reduced by 12% in the vegetation hall (*p* = 0.04), but increased by 26% in the greenhouse compared to control seedlings (*p* < 0.001; Figure 4J). The summer drought had positive effects after stress release on diameter growth from August to November in both buildings (*p* < 0.001, Figure 4J).

### Relation of Growth in 2013 and Drought Response to Aridity at the Origin of Provenances

**Figure 5:**
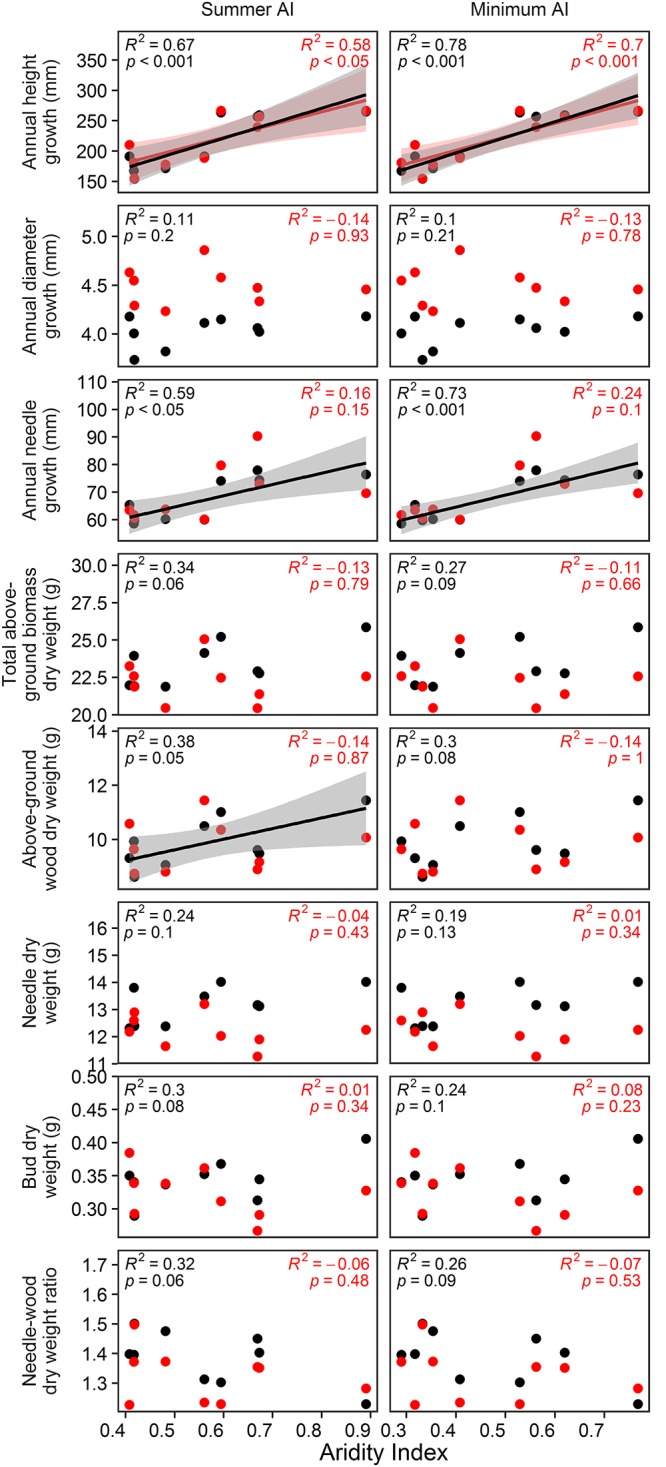
Estimated effects of annual AI, growing season AI, summer AI and minimum AI on annual height, diameter and needle growth as well as on total above-ground biomass, above-ground wood biomass, needle biomass, bud biomass and the needle to wood ratio. Black color indicates the relationships in the vegetation hall whereas red color shows the relationships in the greenhouse. Dots represent respective measured values. D7 was excluded from the analyses, because its AI values exceed the AI values of the second moist site by more than 100%.

In the vegetation hall, annual height and needle growth could be best predicted by minimum AI (*p* < 0.001; Figure 5). In the greenhouse, minimum AI could just predict annual height growth (*p* < 0.001; Figure 5). For biomass parameters, only total above-ground biomass responded to summer AI (*p* = 0.05). For all analyzed parameters, the coefficients of determination decreased when provenances were grown under higher temperatures in the greenhouse.

**Figure 6:**
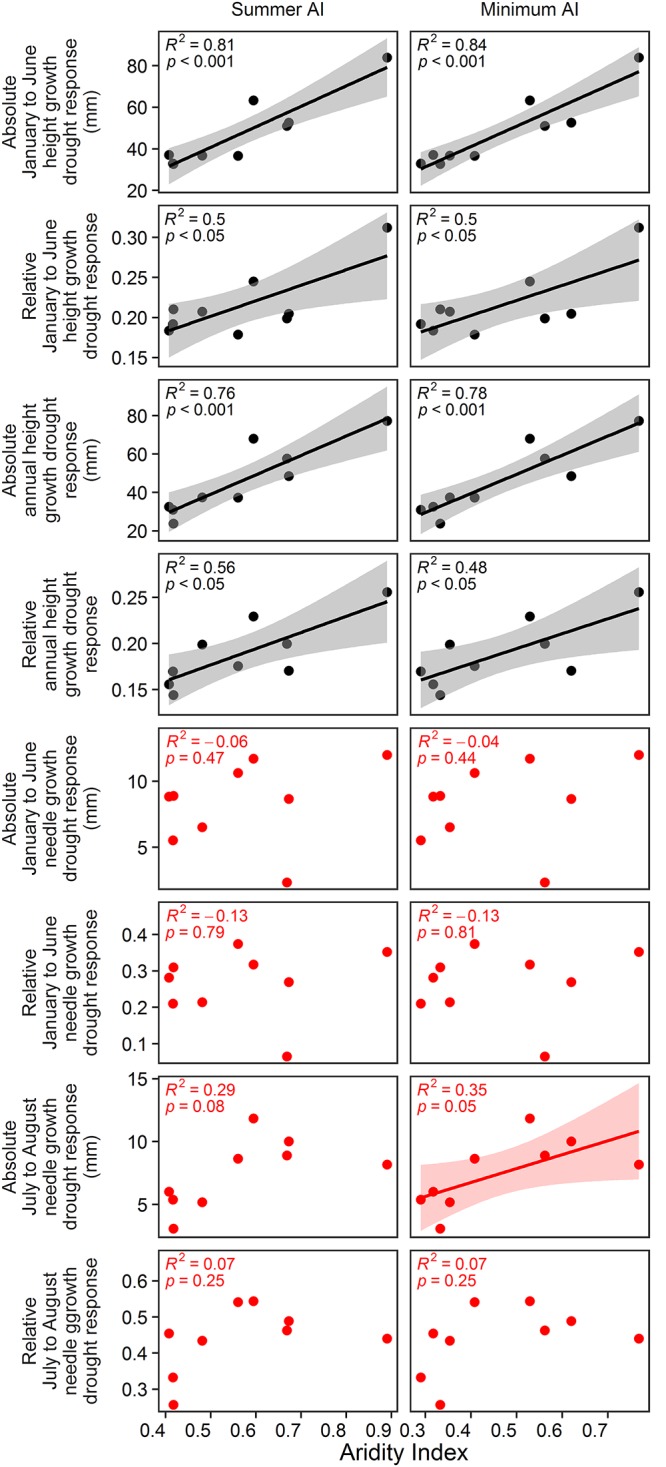
Estimated effects of annual AI, growing season AI, summer AI and minimum AI on absolute and relative drought response of January to June and annual height growth as well as absolute and relative drought response of needle growth from January to June and from July to August. Black color indicates the relationships in the vegetation hall whereas red color shows the relationships in the greenhouse. D7 was excluded from the analyses, because its AI values exceed the AI values of the second moist site by more than 100%.

In the vegetation hall, absolute and relative spring drought response of January to June and annual height growth could be significantly predicted by summer AI and minimum AI (Figure 6; *p* < 0.001 for absolute growth response and *p* < 0.05 for relative growth response). In the greenhouse, only absolute summer drought response of July to August needle growth was significantly explained by minimum AI (Figure 6; *p* < 0.05). Annual AI and growing season AI were not related to any growth, biomass or drought response parameter.

### Phenology in 2014

Mean phenological development in 2014 started 10–14 days earlier in the greenhouse than in the vegetation hall (Supplementary Figure S9). Buds of the southern provenances broke around 2 days earlier than buds of the northern provenances (at least *p* < 0.0013; Supplementary Figures S9A,C and Supplementary Table S6). Previous year spring drought advanced bud break by almost 2 days in the vegetation hall (*p* = 0.018; Supplementary Figure S9B) and by around three and a half days in the greenhouse (*p* < 0.001; Supplementary Figure S9D). The phenophases needle unfolding and needles unfolded responded to current year drought conditions in the vegetation hall, whereas they differed between region and previous year drought conditions in the greenhouse. Therefore, in the vegetation hall, drought advanced needle unfolding (*p* = 0.05) and the day when all needles were unfolded (*p* = 0.018) by more than 1 day (Supplementary Figures S9E,H and Supplementary Table S6). In the greenhouse, southern provenances started to unfold needles (*p* = 0.018) and had needles unfolded (*p* = 0.001) around one and half have days earlier than northern provenances (Supplementary Figures S9F,I and Supplementary Table S6). Finally, the previous year spring and summer drought, respectively, advanced needle unfolding (*p* < 0.001) and needles unfolded (*p* = 0.001) by approximately 2 days.

### Seedling Growth Variation With Provenance, Drought and Phenology in 2014

**Figure 7:**
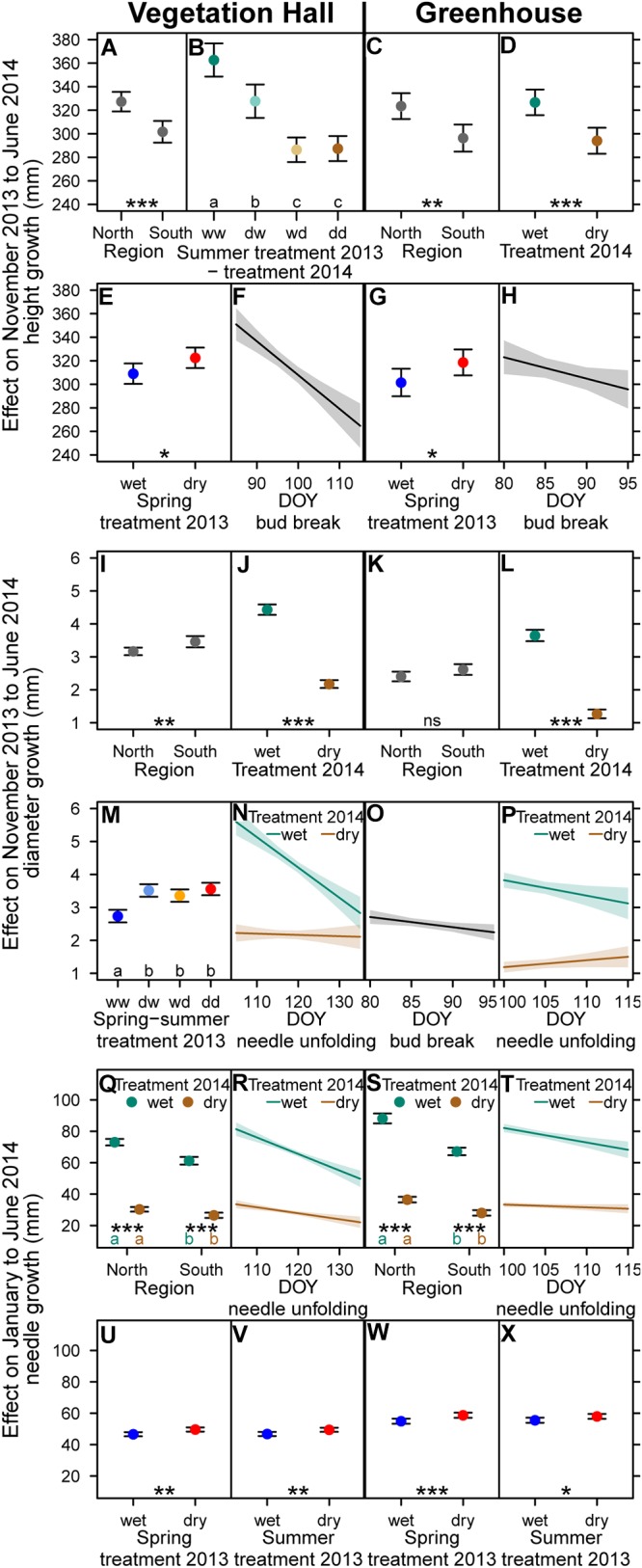
Estimated model effects of explanatory variables included in the final model on **(A–H)** height growth, **(I–P)** diameter growth, and **(Q–X)** needle growth from November 2013 to June 2014 in **(A,B,E,F,I,J,M,N,Q,R,U,V)** the vegetation hall and in **(C,D,G,H,K,L,O,P,S,T,W,X)** the greenhouse. **(E,F)** Height, **(M)** diameter and **(U–X)** needle growth show compensatory growth to previous year drought. Shown are fitted mean values and 95% confidence intervals for each variable holding all other variables constant around their mean. Abbreviations in **(B,M)** denote wet (w) and dry (d) conditions during the drought periods. Regional provenances groups and treatments sharing the same lowercase letter in the same color within buildings are not different at a significance level of 0.05. Levels of significance are ^∗^*p* < 0.05, ^∗∗^*p* < 0.01, ^∗∗∗^*p* < 0.001, ^ns^*p* > 0.05.

Seedlings in both buildings showed similar overall mean height growth, increased overall mean diameter growth (+1.2 mm) but decreased overall mean needle growth (-8,7 mm) in the vegetation hall compared to the greenhouse (Supplementary Tables S7–S9). Northern provenances grew more in height and had longer needles (at least *p* < 0.002; Figure 7A,C,Q,S and Supplementary Tables S7, S9), but grew less in diameter than southern ones (Figure 7I,K and Supplementary Table S8).

Current year drought decreased height and diameter growth (*p* < 0.001) with a stronger decrease of height growth in the vegetation hall than in the greenhouse (Figure 7B,D,J,L and Supplementary Tables S7, S8). The reduction of absolute needle growth by current year drought was stronger in the northern provenances than in the southern provenances (Figure 7Q–T, Supplementary Table S9 and Supplementary Figure S10).

Carryover drought effects of the previous year summer drought still reduced height growth of well-watered seedlings in the vegetation hall (-10% or -35 mm; *p* < 0.001) (Figure 7B and Supplementary Table S7).

Positive carryover effects of the previous year spring drought and additionally of the previous year summer drought were apparent for height and needle growth, respectively (Figure 7E,G,U–X and Supplementary Tables S7, S9). Furthermore, in the vegetation hall, seedlings that experienced at least one drought event in the previous year grew 23–30% more (*p* < 0.001) in diameter in 2014 (Figure 7M and Supplementary Table S8).

Later bud break resulted in lower height growth in both buildings (Figure 7F,H and Supplementary Table S7) and in slightly lower diameter growth in the greenhouse (*p* = 0.02; Figure 7O and Supplementary Table S8). Later onset of needle unfolding resulted in lower diameter growth and shorter needles under well-watered conditions (Figure 7N,P,R,T and Supplementary Tables S8, S9). In general the phenological effects on height and needle growth were more pronounced in the vegetation hall than in the greenhouse.

### Influence of Provenance and Drought on Quantum Efficiency of Photosystem II and Stable Carbon Isotope Ratio

Quantum efficiency of PSII during the summer 2013 was not influenced by the actual drought in the vegetation hall, but was lower under drought in the greenhouse (*p* < 0.001; Supplementary Figure S11A and Supplementary Table S10). In the vegetation hall, previous spring drought had a positive effect on quantum efficiency (*p* < 0.001), whereas in the greenhouse quantum efficiency was still negatively affected (*p* = 0.04; Supplementary Figure S11A). The provenances I4, ES1, and F3 had the lowest quantum efficiency during the summer treatment period (Supplementary Figure S12).

In the vegetation hall after summer drought stress release, previous spring or summer drought experience increased quantum efficiency (at least *p* < 0.02; Supplementary Figure S11B and Supplementary Table S10). In the greenhouse, summer drought reduction of quantum efficiency still persisted after drought stress release (*p* = 0.001, Supplementary Figure S11B and Supplementary Table S10). In 2014, southern provenances had lower quantum efficiencies than northern provenances (*p* < 0.008). Overall, quantum efficiency was significantly reduced (*p* < 0.001) by drought (Supplementary Figure S13 and Supplementary Table S11).

The differences of stable carbon isotope ratios between provenances and treatments were more distinct in the greenhouse than in the vegetation hall, although stable carbon isotope ratio followed the same patterns (Supplementary Figure S14 and Supplementary Table S12). Therefore, the provenances D8 and BG10 showed less carbon discrimination than the provenances I4 and ES1 (Supplementary Figure S14). A reduction of carbon discrimination was caused by summer drought and was intensified by spring drought (Supplementary Figure S12).

### Association Between Physiological and Structural Response

**Figure 8:**
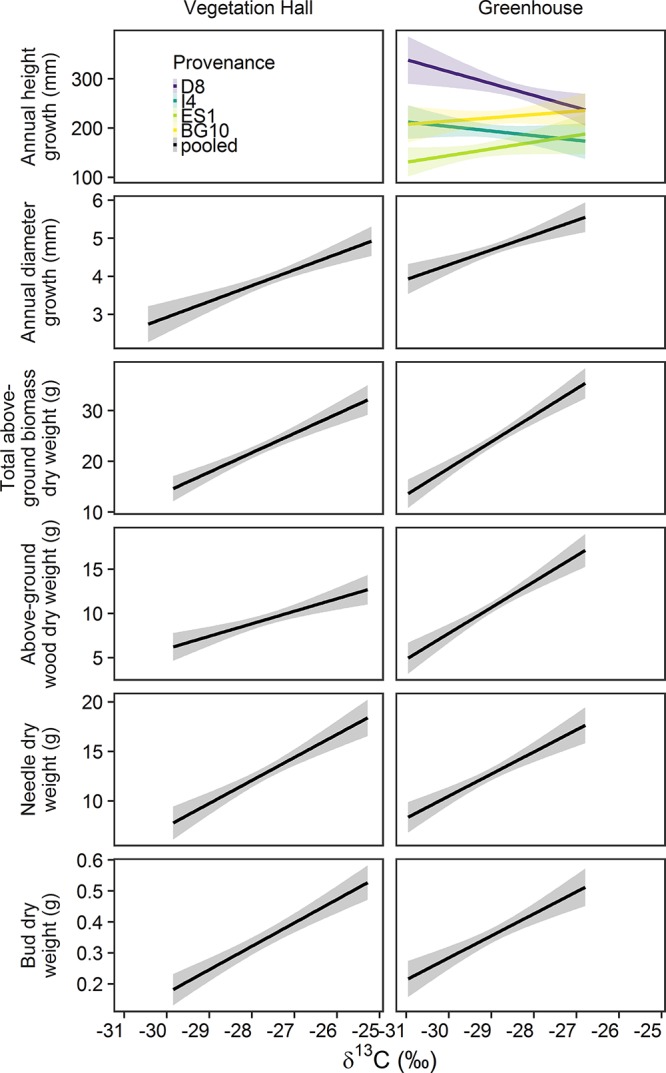
Estimated model effects of the stable carbon isotope ratio (δ^13^C) on annual height and needle growth, total above-ground biomass, above-ground wood biomass, needle biomass and biomass in the vegetation hall and the greenhouse. Note that δ^13^C could not explain annual height growth in the vegetation hall. Provenance abbreviations are D8 (Mittel-/Ostdt. Tiefland, Germany), I4 (Emilia Romagna, Italy), ES1 (Alto Ebro, Spain), and BG10 (Garmen, Bulgaria).

Annual diameter growth and biomass showed a positive relationship with δ13C while accounting for drought effects (Figure 8 and Supplementary Tables S13, S14). In the vegetation hall, annual height growth was not associated with stable carbon isotope ratio, whereas in the greenhouse, this association varied between provenances (Figure 8 and Supplementary Tables S13, S14). Height growth of D8 and I4 decreased, while height growth of ES1 and BG10 increased with rising stable carbon isotope ratio. Drought treatments generally had a negative influence on growth and biomass (Supplementary Tables S13, S14). Since results of drought and provenance influences on growth and biomass were similar to the results described in “Annual variations of seedling growth and wood, needle and bud biomass in 2013”, we kindly refer to this chapter for more detailed information.

## Discussion

### Provenance Effects

Phenology, growth traits, biomass and carbon isotope ratios differed between provenances, indicating genotypic variation. An analysis of isozymes of most of the provenances used in our experiment found differences in genetic variability and genetic diversity (Taeger et al., 2013a). Provenance-specific growth patterns could thus be a reflection of adaptations to the climatic conditions at their origins, especially since site aridity index and diverse growth parameters were positively correlated. Respectively, various drought growth responses were lower in provenances from drier origins especially when assessed in absolute terms.

Onset of phenophases in 2013, particularly of needle unfolding and needles unfolded in the vegetation hall, occurred earlier in northern than in southern provenances, agreeing with results from a previous study by Taeger et al. (2015). Generally, forcing requirements can differ between populations (Hannerz et al., 2003) and provenances from colder origins often require lower temperature sums to trigger bud break (Berninger, 1997). However, this pattern could not be confirmed in our greenhouse experiment of spring 2013, most likely because temperatures never fell below 0°C, causing higher forcing requirements when the natural chilling requirements of *P. sylvestris* were not met (Laube et al., 2014). In contrast, onsets of phenophases in 2014 were earlier for southern than for northern provenances. All provenances in the southern group of 2014 originated from high elevations with cooler mean spring temperatures than those at the origins of the northern group (Seidel and Menzel, 2016). This difference between low and high elevation populations may be comparable to the differences between southern and northern latitude populations. The results of phenology in 2014 can be confirmed by additional analyses of phenophases in 2013 with the same groups of provenances as used in 2014 (results not shown).

Southern provenances generally showed smaller height and needle growth, but also a smaller drought-induced reduction of height growth and needle growth in 2013 and 2014, respectively. Oleksyn et al. (1992, 1998, 1999) and Semerci et al. (2017) found similar differences in overall height and needle growth between northern and southern provenances. The provenance-specific height and needle growth suggests a lower phenotypic response but also a better adaptation to drought by individuals from southern provenances. This is supported by our finding that height and needle growth is positively correlated with aridity at the site of their origin. Since tree height and needle area are positively correlated (Xiao and Ceulemans, 2004; Jagodziński and Kałucka, 2008), the reduction of stem and needle growth itself might decrease evaporative water loss and thus increase resistance to drought. Additionally, smaller trees have a lower risk of suffering hydraulic dysfunction because of physical and anatomical aspects. Forces to lift water in xylem conduits against gravity and conduit resistance are lower in smaller sized trees, reducing the risk of a water column collapse and thus the impairment of water transport by xylem cavitation (McDowell and Allen, 2015). Furthermore, conduit size increases with the distance to the stem tip (Anfodillo et al., 2006, 2013; Olson et al., 2014), resulting in a higher risk of xylem cavitation (Martínez-Vilalta et al., 2009; Sterck et al., 2012). It has been shown on a global scale and for Scots pine seedlings in particular, that tree height is linked to a higher risk of drought-induced mortality (Bennett et al., 2015; Seidel and Menzel, 2016). The drought adaption of southern provenances is also reflected in the stable carbon isotope ratios of needles, similar to *Pinus pinaster* (Correia et al., 2008). Southern provenances (I4, ES1, and BG10) show more negative values than D8, suggesting lower water use efficiency (Jones, 2013), higher maximum net photosynthesis (DeLucia and Schlesinger, 1991) and higher stomatal conductance (DeLucia et al., 1988) during carbon fixation. This loose stomatal control might be of advantage in arid climates since photosynthesis can continue for longer periods. Moreover, ES1 and BG10 which originate from drier sites than D8 and I4, can translate higher water use efficiency to higher height growth, whereas higher water use efficiency in D8 and I4 is linked to reduced height growth. This might further suggest provenance-specific adaptation to drought, since a positive relationship between water use efficiency and height growth could indicate higher assimilation, while a negative relationship would indicate stronger stomatal control (Marguerit et al., 2014). A looser stomatal control of the southern provenances, and thus a partial exploitation of water resources in pots, might also be seen through the slightly lower values of photosynthetic efficiency we recorded in 2014.

A provenance-specific drought response of height growth was detectable in the vegetation hall for the period from January to June and for the annual period in 2013, but not from November 2013 until June 2014. In a provenance trial in Poland (Oleksyn et al., 2001) duration of Scots pine shoot elongation was about 60–70 days and reached its maximum growth rate after 42–48 days. In our experiment, the time span between bud break and maximum drought (soil moisture below the permanent wilting point) ranged between 36 and 40 days (greenhouse in 2013, during 2014) and just 13 days for the vegetation hall in 2013. The height growth of seedlings in the vegetation hall in 2013 was thus affected by intensive drought for a much longer time, while seedlings in all other cases could have partially escaped drought impacts, diluting our observations of provenance-specific drought responses. Experiencing higher drought stress exposure could also be an explanation for the provenance-specific differences in drought response, e.g., of needle growth. Higher temperatures in the greenhouse increased the mean and maximum vapor pressure deficit by 0.18–0.35 kPa and 1.2–1.45 kPa, intensifying stress during the spring and summer drought; in contrast to 2013, the drought treatment groups of 2014 did not receive any water at all during the drought period, leading to soil moisture values well below the species’ permanent wilting point for several weeks.

Diameter growth and biomass parameters also differed between provenances, but without as clear of a pattern as height and needle growth. Most prominent differences include the superiority of Alpenkiefer (D7) and Plantage Pornoapati (HU14), whose seeds came from seed orchards aiming at profitable growth and biomass production. The high variability of provenances in biomass production was also confirmed by other authors (Oleksyn et al., 1999, 2000), however, their reported relationship of total above-ground biomass with latitude was driven by a broader latitudinal range than in our study. This may imply that diameter growth is not under a pronounced climate-related selective pressure compared to height and needle growth, as indicated by our study results. Lastly, we found no pattern between needle dry weight and needle growth. This disagreement might be explained by the observation that specific leaf area is variable among individuals from the same provenance and does not follow a particular pattern (Taeger et al., 2015). Additionally, there are differences in biomass allocation to above-ground compartments between trees grown under non-drought and drought conditions (DeLucia et al., 2000).

Thus, in accordance with recent literature findings, our results strongly suggest that provenances differ in their drought response especially with regards to phenology, height and needle growth as well as stable carbon isotope ratio (research question 1b, c). Some of these differences were linked to their climatic origin assuming local adaptation. However, these relationships of growth and biomass parameters diminished under warmer conditions in the greenhouse, which might ultimately hinder the selection of provenances suitable for assisted migration with ongoing climate change.

### Drought and Building Effects

Direct drought effects, i.e., the response of Scots pine seedlings to drought, are manifold (research question 1a): seasonal drought treatments directly affected almost all phenological, growth and ecophysiological traits except bud break in 2013, phenophases in the greenhouse in 2014 and quantum efficiency of the PSII in the vegetation hall in 2013. We mainly attribute building effects to the higher temperatures measured in the greenhouse consequently increasing the mean and maximum vapor pressure deficit by 0.33 and 1.4 kPa during frost free periods. This higher evaporative demand has probably caused more distinct differences of carbon discrimination between provenances in the greenhouse. However, radiation could be controlled by automated shading to prevent over-heating of the greenhouse in summer what might have caused higher needle carbon isotope ratios induced by reduced radiation (Brendel et al., 2003).

Current year drought delayed the phenological development of buds and leaves in 2013, but advanced it in 2014. The delay of phenology might be due to low water potentials impeding tissue formation (Hsiao and Acevedo, 1974; Peñuelas et al., 2013). Bernal et al. (2011) also observed advanced spring phenology during drought and attributed it to a lack of transpirational cooling and thus earlier phenological development. This mechanism might be constrained by the reduced growth caused by low water potentials. In the vegetation hall, soil moisture was lower and mean and maximum vapor pressure deficits were 0.1 and 0.8 kPa higher during the mean onset of phenophases in 2013 than in 2014, suggesting higher drought stress during phenological development in 2013. This might have caused the switch from drought-induced advance to drought-induced delay of phenology. Spring phenology in the greenhouse, apart from needles unfolded in 2013, did not respond to the current year drought treatments since the mean onset of phenophases occurred before the severe drought conditions around the permanent wilting point. Depending on the phenophase, onset dates were 10–20 days earlier in the greenhouse, matching the well-known advance of phenology with higher temperature (Reyer et al., 2013).

Growth reduction under drought might not be due to a limitation of photosynthesis since tissue growth commonly decreases ahead of carbon assimilation (Muller et al., 2011), indicating a sink rather than a source limitation (Körner, 2015); a drought-related growth decline of Scots pine has been observed even though the pool of carbon assimilates increased (Gruber et al., 2011; Bachofen et al., 2017). Drought generally decreases turgor pressure or induces the production of growth regulators, which in turn reduces cell division and expansion (Hsiao and Acevedo, 1974; Peñuelas et al., 2013). Nevertheless, our findings show that drought increased the stable carbon isotope ratio and decreased the quantum efficiency of the PSII, suggesting stomatal closure along with a limitation of the PSII, thereby restricting photosynthesis (DeLucia et al., 1988; Ashraf and Harris, 2013).

A lacking influence of summer drought on quantum efficiency of the PSII in the vegetation hall in 2013 can be explained by drought severity. Since the mean and maximum vapor pressure deficit was 0.35 and 1.2 kPa lower than in the greenhouse, the stress for seedlings was smaller (Williams et al., 2012). Thus, drought stress in summer 2013 in the vegetation hall might have been too low to induce an inhibition of PSII, as suggested in a similar study on Norway spruce (Pukacki and Kamińska-Rożek, 2005).

Drought conditions increased the needle to wood dry weight ratio indicating that needle growth was less sensitive to drought than wood growth. This finding is contradictory to other studies since the ratio between transpiring and water transporting tissue should decrease with drier conditions (Poyatos et al., 2007; Martínez-Vilalta et al., 2009) and it may not solely be related to different life stages (young vs. adult) or study conditions (experiment with potted individuals vs. field studies).The reduction of a considerable amount of foliage can be a long lasting process (Dobbertin, 2005), whereas the adjustment of the xylem can be very fast (Bryukhanova and Fonti, 2013); thus, we might have missed the ultimate drought impact on needle to wood biomass ratio since we only measured biomass during the 1st year of drought.

Elevated temperatures have been shown to decrease height and diameter growth of mature Scots pine in the field (Martínez-Vilalta et al., 2008; Reich and Oleksyn, 2008; Michelot et al., 2012), but there was no warming effect in Scots pine and Ponderosa pine seedling experiments (Maherali and DeLucia, 2000; Taeger et al., 2015), confirming a change of sensitivity to environmental influences with ontogeny (Niinemets, 2010). In our study we did not find obvious differences in height growth between the cooler vegetation hall and the warmer greenhouse, although in a previous study height growth of PL9, D7, and F12 was lower in the greenhouse than in the vegetation hall (Seidel and Menzel, 2016). If the reduced set of provenances had been considered in our current study, the results presented here would be similar (data not shown). The sensitivity of trees to environmental influences can change with ontogeny and thus alter their responses to climatic conditions (Niinemets, 2010). In contrast to temperature-insensitive height growth, diameter growth under warmer conditions in the greenhouse in 2013 was higher than under cooler temperatures in the vegetation hall, although this pattern was reversed in 2014. The overall differences in annual diameter growth in 2013 matched the increased growth from August to November, suggesting a longer growth period induced by higher temperatures (Peltola et al., 2002; Rossi et al., 2014). Spring radial growth in the greenhouse in 2014 might have been constrained by the depletion of carbohydrate reserves during the longer growing period in 2013. Early wood formation relies on stored carbohydrates, which are affected by late wood formation in the previous year (Oberhuber et al., 2011).

Compared to the existing literature, our study was unique in showing interactive effects of previous drought events, either in the same year (spring summer) or in the subsequent year (2013 and 2014) on seedling response (see research question 2). For several parameters we could show that previous drought experience reduced the impact of a following extreme event.

### Carryover Effects and Compensation

Related to our third research question, the results clearly revealed drought-related carryover effects in the greenhouse for (a) intra-annual diameter growth, (b) for the efficiency of PSII and (c) in the vegetation hall for inter-annual shoot growth. Naturally, recovery of water potentials takes longer under warmer conditions (Balducci et al., 2016), prolonging the time to reach the necessary cell turgor pressure for cell division and expansion (Hsiao and Acevedo, 1974) and thereby inhibiting diameter growth, as might have been the case after our spring drought in 2013. Most likely, different timings of bud set in the greenhouse and in the vegetation hall modulated the influence of the summer drought in 2013 on shoot growth in 2014. Notably, current year shoot growth potential in Scots pine is related to previous year conditions during bud formation (Wareing, 1956; Junttila and Heide, 1981) as well as to water availability during the previous summer (Jansons et al., 2015). Elevated temperatures for example, can delay bud dormancy and bud formation (Wareing, 1956; Strømme et al., 2016). Taeger et al. (2013a) showed that 50% of bud set under greenhouse conditions was achieved in September. Consequently, in our study, bud set might have occurred earlier in the vegetation hall and overlapped with the summer drought, thereby reducing growth in the following year. The efficiency of PSII was obviously not recovered after the more severe spring and summer drought in the greenhouse, indicating damage of the PSII reaction centers (Ashraf and Harris, 2013). Following drought stress release in the vegetation hall, efficiency of PSII was higher in drought stressed than in non-stressed seedlings. We assume that one of the drivers of increased photoprotection was triggered during the drought treatment and led to an increased efficiency of the PSII once seedlings were well-watered (Derks et al., 2015).

Phenology in 2014 was advanced by previous year drought when not impaired by current year water availability. This could be beneficial for Scots pine by escaping unfavorable conditions during spring. Similar results have been documented for various oak species (Spieß et al., 2012; Kuster et al., 2014). Since environmental conditions during bud formation influence succeeding year growth (Wareing, 1956; Junttila and Heide, 1981) it is likely that phenological development could also be affected, although we are not aware of any study describing a mechanism behind earlier phenology induced by previous year drought.

Pine seedlings responded with intra- and inter-annual compensatory growth of height, diameter and needles after drought stress release, thus affirming our research question 3. Enhanced shoot growth upon re-watering after drought was observed in *Quercus petraea* within and across years (Spieß et al., 2012; Turcsán et al., 2016). Arend et al. (2016) as well as Pflug et al. (2018) observed stimulated net-photosynthesis in formerly drought stressed *Fagus sylvatica* saplings until the end of the vegetation period, partly counterbalancing previous drought effects. Numerous studies show an accumulation of non-structural carbohydrates when water availability is low (Gruber et al., 2011; Muller et al., 2011; Bachofen et al., 2017; Piper et al., 2017). Since phloem transport of non-structural carbohydrates can be impaired under insufficient water supply (Sala et al., 2010), the release from drought stress might induce changes in carbon allocation from source to sink tissue and thus provoke an increased growth rate. This is in line with recent findings, which show that the non-structural carbon pool size is positively related to ring-width growth (von Arx et al., 2017). Allocation dynamics of non-structural carbon to different organs varies during the year (Rosas et al., 2013), indicating that drought timing may influence the growth response. This can be clearly seen in our study for the inter-annual response of height growth in relation to the previous year spring and summer drought. Nevertheless, compensatory growth might differ between ontogenetic stages since the ratio between currently assimilated carbon and carbon pools is higher in seedlings compared to mature trees (Niinemets, 2010).

## Conclusion

In this study we demonstrate that Scots pine seedlings show a highly plastic (direct) response of phenology, growth and ecophysiological parameters to reoccurring drought events. Our results suggest that intra-annual compensatory growth, however, is not sufficient to fully offset the drought-induced reduction of annual growth, and can only help to mitigate these impacts. Nonetheless, we were able to identify carryover effects and show that this compensatory growth can also occur on an inter-annual time scale. Interactive effects of multiple droughts may have the ability to render seedlings resistant against negative direct impacts. Additionally, we were able to show that the timing of drought, in relation to phenology, modulates the influence on seedling growth and phenology itself. Lastly, our findings suggest that southern provenances of Scots pine are better adapted to drought conditions than northern ones; the former display a less severe drought response and exhibit morphological characteristics associated with drought resistance. Although southern provenances appear to be less productive as a result of lower height and needle growth, it may actually render them more resilient to extreme climatic events and highlights the apparent trade-off between productivity and drought resistance. The predictability of provenances’ drought performance through climatic parameters might nonetheless be constrained by higher temperatures in the future. Warmer temperatures could counteract plastic responses due to intensified drought conditions and shifting phenophases, leading to indirect drought effects. More studies are needed to better understand the relationship between carbon assimilation, allocation, storage and use during and after drought conditions, a hot topic in current climate change and forestry research (Granda et al., 2017). The influence of drought timing and phenology on inter- and intra-annual tree growth should also be further investigated.

## Author Contributions

HS collected data, contributed to the experimental design, analyzed and interpreted the data, and wrote the manuscript. MM contributed to data analyses and interpreted the data. AM contributed to the conception of the work, interpreted the data, and wrote the manuscript.

## Conflict of Interest Statement

The authors declare that the research was conducted in the absence of any commercial or financial relationships that could be construed as a potential conflict of interest.
