# Supplementary material for: Compensatory Growth of Scots Pine Seedlings Mitigates Impacts of Multiple Droughts Within and Across Years
Source: Front Plant Sci. 2019 Apr 24;10:519. doi: 10.3389/fpls.2019.00519 (PMC6491932; doi:10.3389/fpls.2019.00519)
Supplement: Supplementary file 1 [file Table_1.DOCX]

Supplementary Material

**Compensatory growth of Scots pine seedlings mitigates impacts of multiple droughts within and across years**

**Hannes Seidel^1*^, Michael Matiu^1,2^, Annette Menzel^1,3^**

^1^Professorship of Ecoclimatology, Department of Ecology and Ecosystem Management, TUM School of Life Sciences Weihenstephan, Technische Universität München, Germany

^2^Institute for Earth Observation, EURAC Research, Bolzano, Italy

^3^Institute for Advanced Study, Technische Universität München, Garching

*** Correspondence:** hseidel@wzw.tum.de

# Supplementary Figures and Tables

## Supplementary Figures


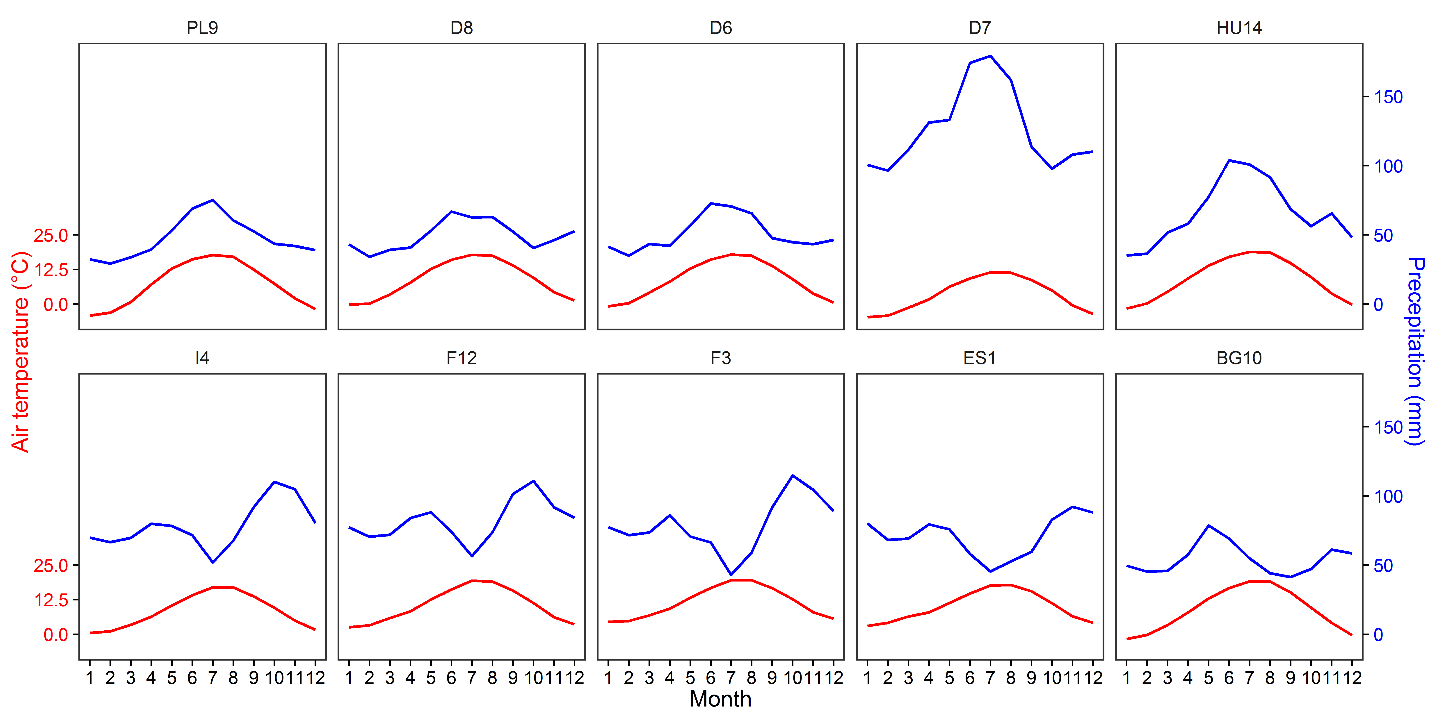
Supplementary Figure 1. Climatograms from the origin of provenances. Climate data obtained from the Climate Research Unit (CRU TS 4.02; Harris et al., 2014) for the period 1950 – 2000. Provenance abbreviations are PL9 (Suprasl, Poland), D8 (Mittel-/Ostdt. Tiefland, Germany), D6 (Hauptsmoorwald, Germany), D7 (Alpenkiefer, Germany), HU14 (Plantage Pornoapati, Hungary), I4 (Emilia Romagna, Italy), F12 (Mont Ventoux, France), F3 (Prealpes du Sud, France), ES1 (Alto Ebro, Spain) and BG10 (Garmen, Bulgaria).


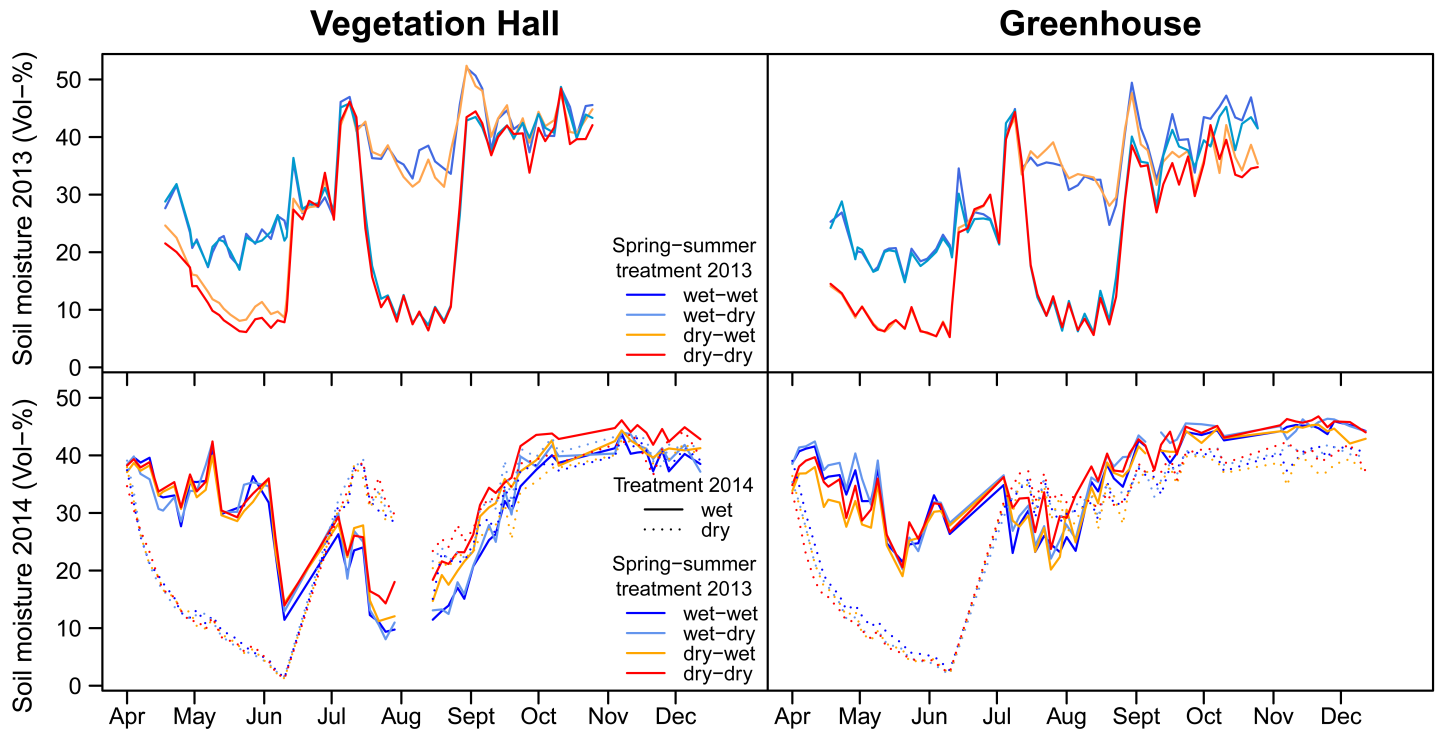


Supplementary Figure 2. Soil moisture development for the different treatments in the vegetation hall and the greenhouse during 2013 and 2014. Note that due to malfunction of the automated dripping system in the vegetation hall in July 2014 the soil moisture in the control group fell below the soil moisture of the drought treatment group. Analysis of morphological and ecophysiological traits was therefore just done till the end of the drought period on June 23, 2014.


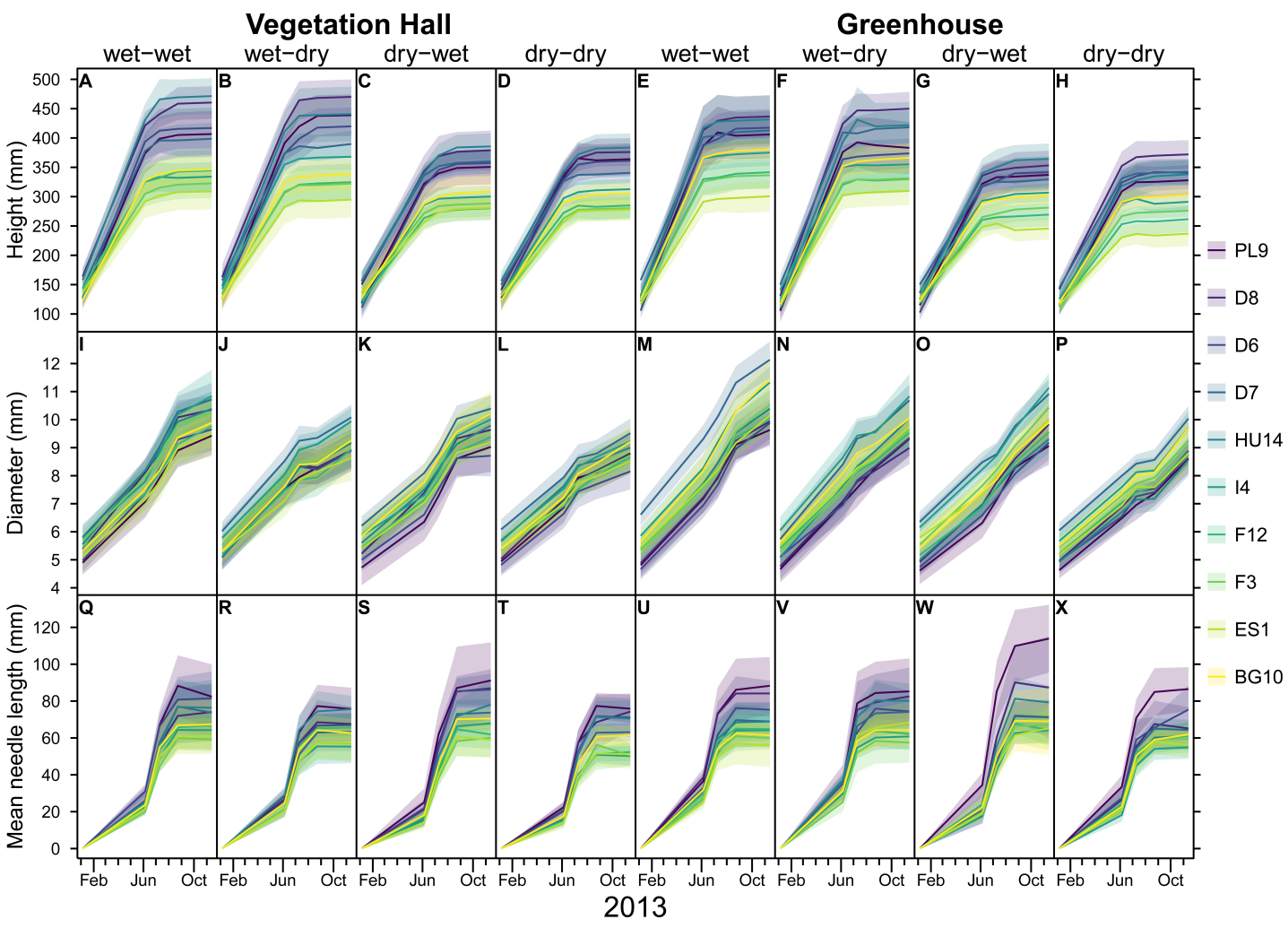


Supplementary Figure 3. Development of (A-H) height, (I-P) diameter and (Q-X) mean needle length of provenances in the control (wet-wet), spring drought (dry-wet) or/and summer drought (wet-dry/dry-dry) treatment groups in the vegetation hall and in the greenhouse from January to November 2013. Mean values and 95% confidence intervals are shown. Provenance abbreviations are PL9 (Suprasl, Poland), D8 (Mittel-/Ostdt. Tiefland, Germany), D6 (Hauptsmoorwald, Germany), D7 (Alpenkiefer, Germany), HU14 (Plantage Pornoapati, Hungary), I4 (Emilia Romagna, Italy), F12 (Mont Ventoux, France), F3 (Prealpes du Sud, France), ES1 (Alto Ebro, Spain) and BG10 (Garmen, Bulgaria).


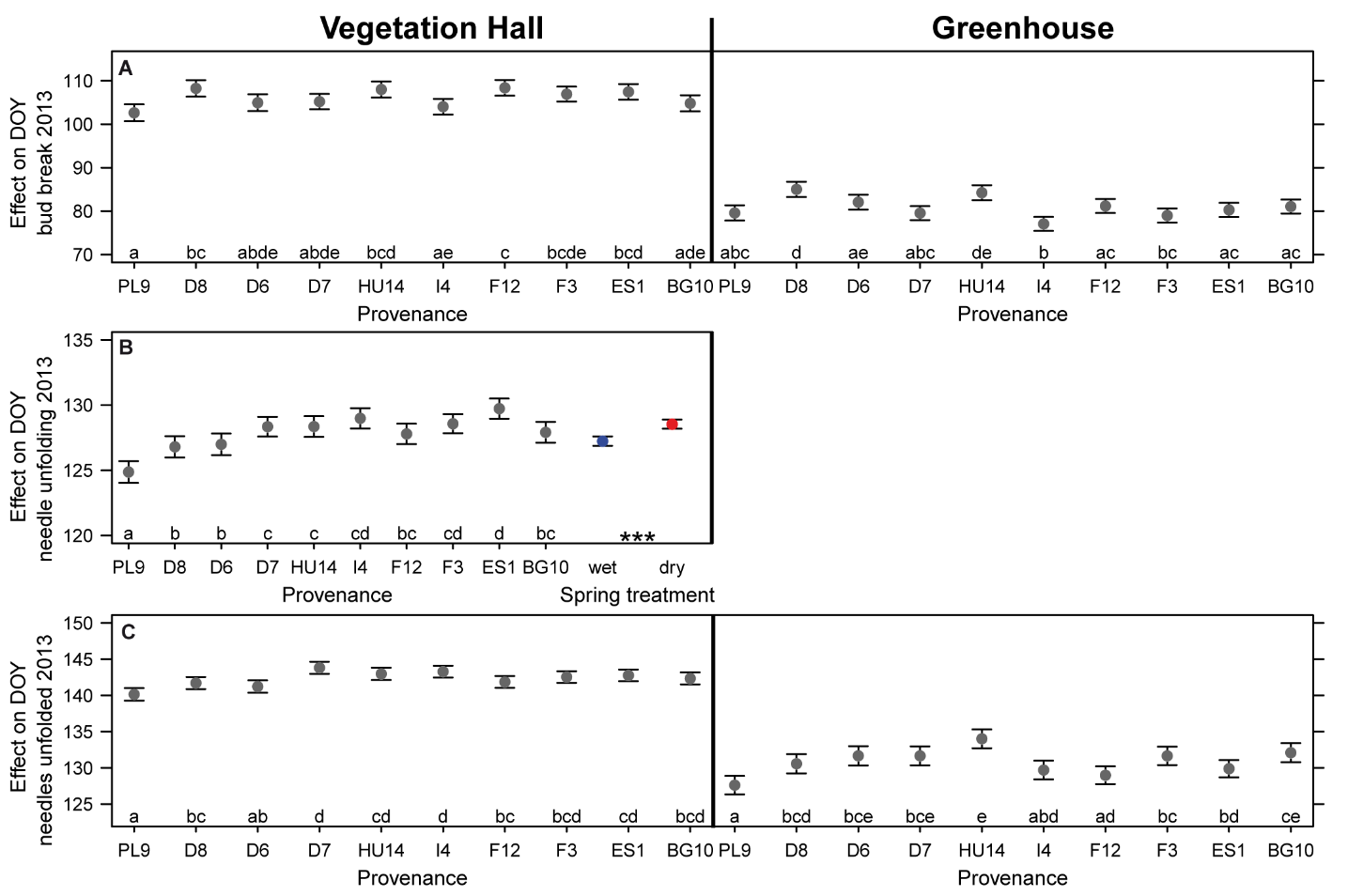


Supplementary Figure 4. Estimated model effects of provenance and spring drought treatment on the onset of (A) bud break, (B) needles unfolding and (C) the day when needles were unfolded in 2013. Shown are fitted mean values and 95% confidence intervals holding all other variables included in the final model constant around their mean. Provenances sharing the same lowercase letter within each building are not different at a significance level of 0.05. Significance levels of asterisk are *** *p* < 0.001. Provenance abbreviations are PL9 (Suprasl, Poland), D8 (Mittel-/Ostdt. Tiefland, Germany), D6 (Hauptsmoorwald, Germany), D7 (Alpenkiefer, Germany), HU14 (Plantage Pornoapati, Hungary), I4 (Emilia Romagna, Italy), F12 (Mont Ventoux, France), F3 (Prealpes du Sud, France), ES1 (Alto Ebro, Spain) and BG10 (Garmen, Bulgaria).


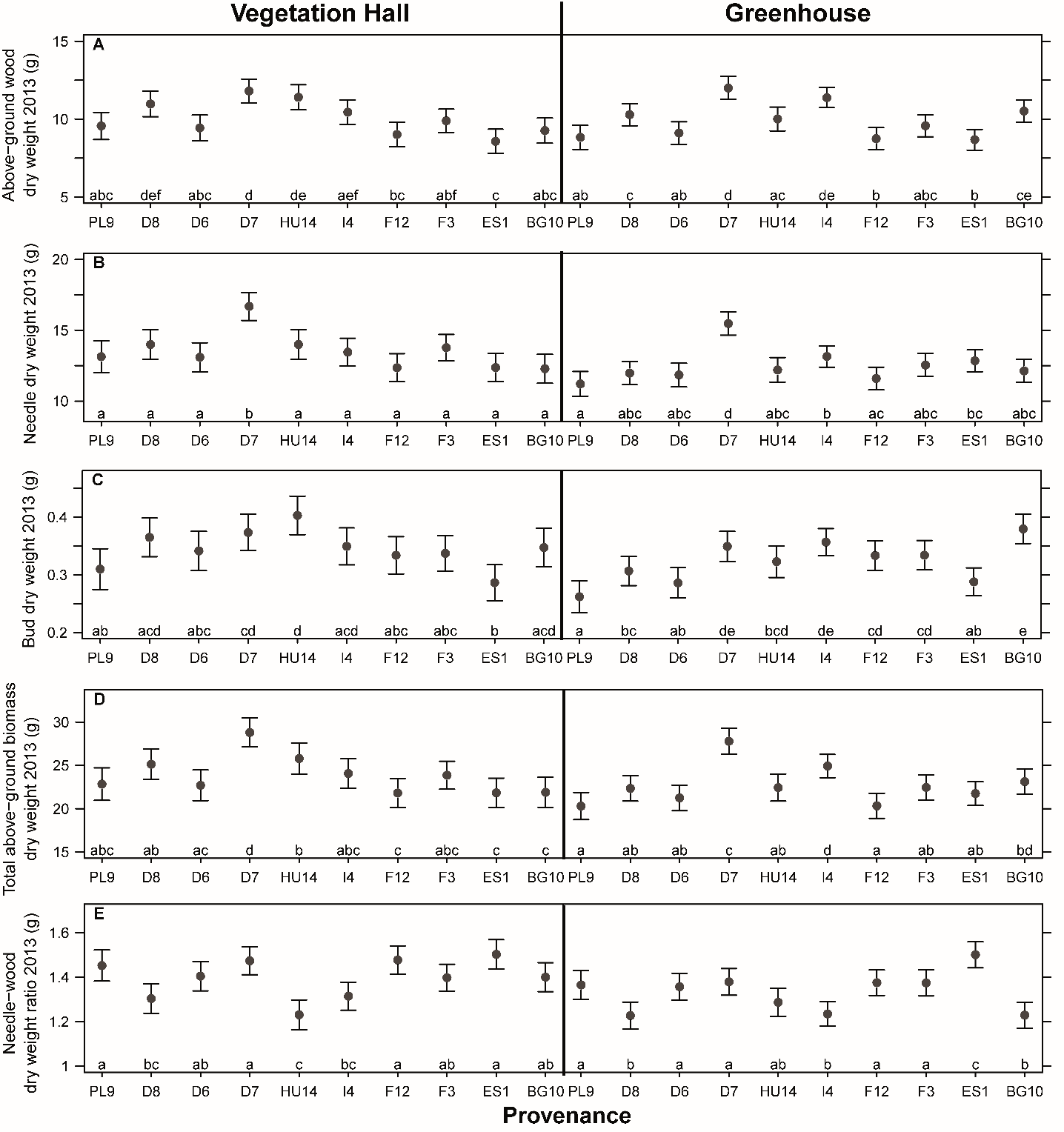
Supplementary Figure 5. Estimated model effects of provenance on (A) above-ground wood dry weight, (B) needle dry weight, (C) bud dry weight (D) total above-ground biomass dry weight and (E) needle to wood dry weight ratio in 2013. Shown are fitted mean values and 95% confidence intervals holding all other variables included in the final model constant around their mean. Provenances sharing the same lowercase letter within each building are not different at a significance level of 0.05. Provenance abbreviations are PL9 (Suprasl, Poland), D8 (Mittel-/Ostdt. Tiefland, Germany), D6 (Hauptsmoorwald, Germany), D7 (Alpenkiefer, Germany), HU14 (Plantage Pornoapati, Hungary), I4 (Emilia Romagna, Italy), F12 (Mont Ventoux, France), F3 (Prealpes du Sud, France), ES1 (Alto Ebro, Spain) and BG10 (Garmen, Bulgaria).


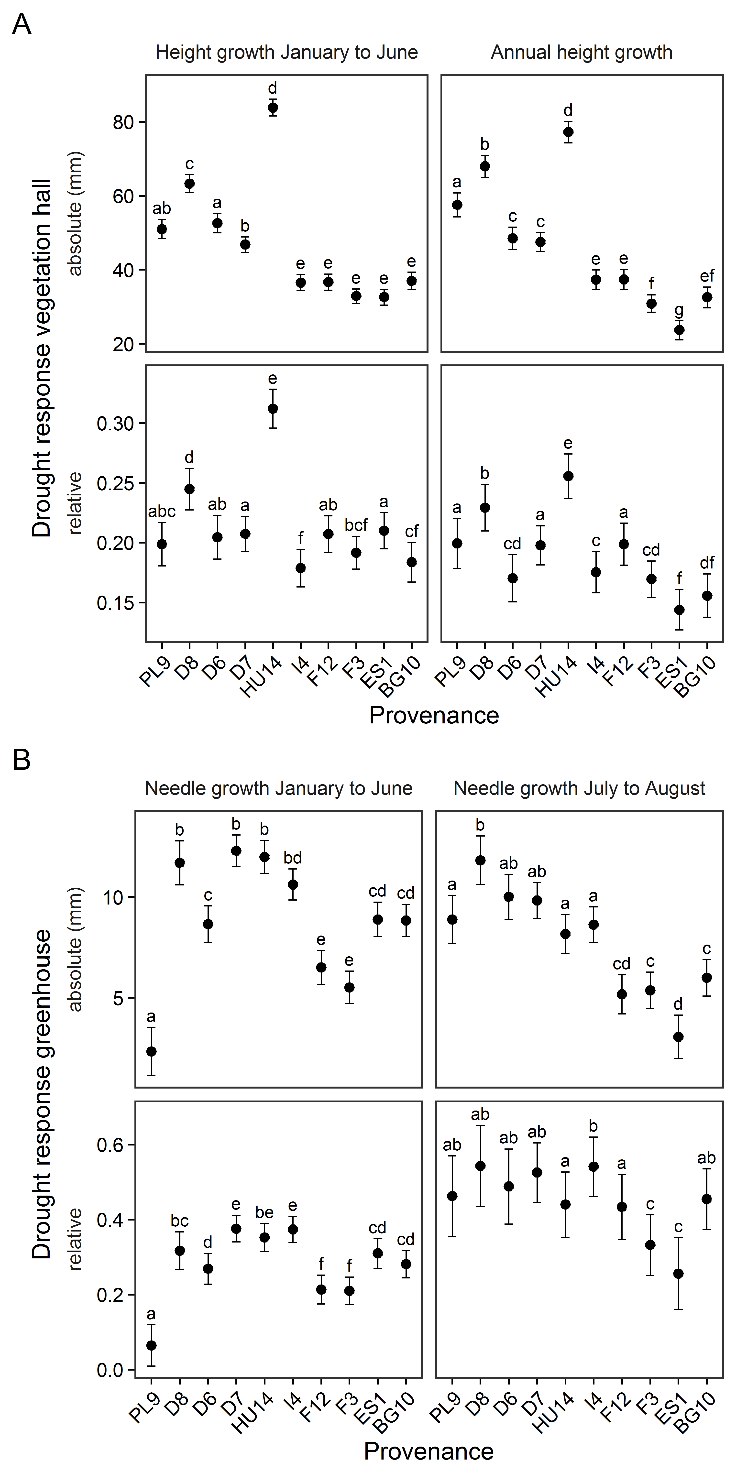


Supplementary Figure 6. Absolute and relative response of (A) height growth from January to June and annual height growth in the vegetation hall to the spring drought 2013 as well as of (B) needle growth from January to June and from July to August in the greenhouse to spring and summer drought 2013, respectively. Higher values indicate a stronger drought response. Differing letters within each panel indicate significant differences between provenances at a level of at least 0.05.


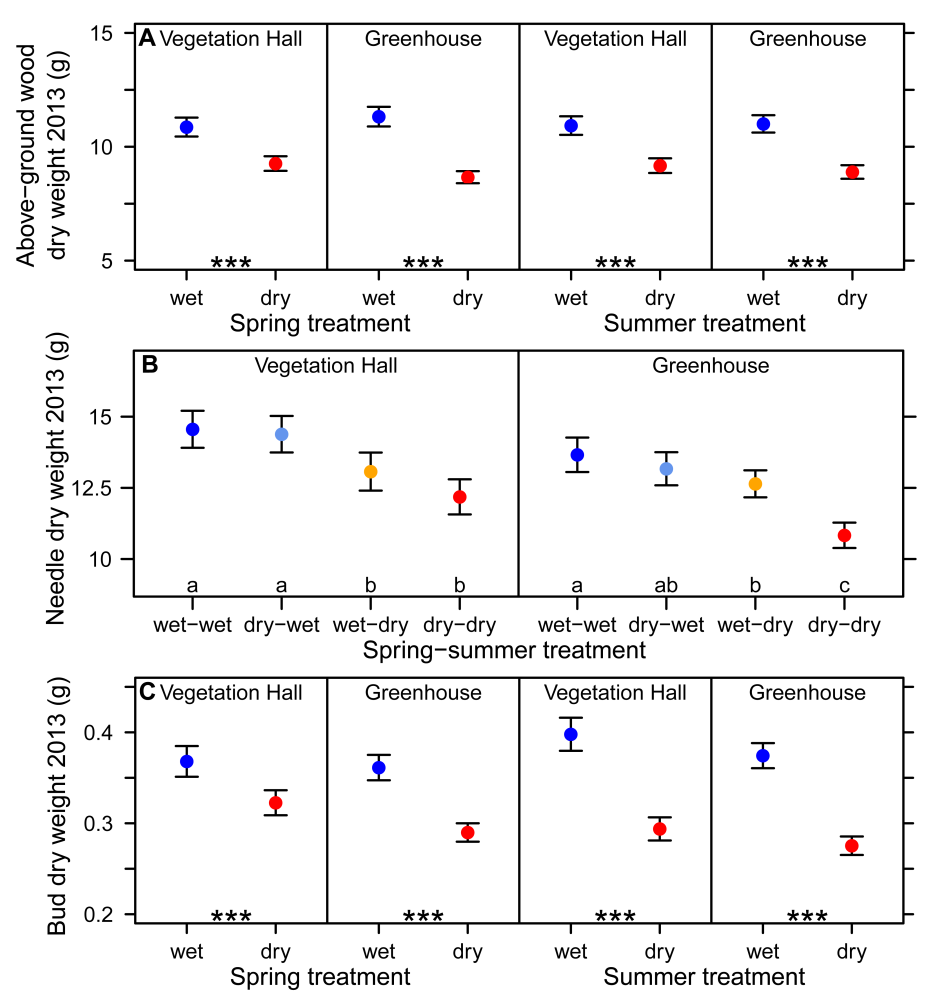
Supplementary Figure 7. Estimated model effects of the spring drought and the summer drought treatment on (A) above-ground wood dry weight, (B) needle dry weight and (C) bud dry weight in 2013. Shown are fitted mean values and 95% confidence intervals holding all other variables included in the final model constant around their mean. Significance levels of asterisk are *** *p* < 0.001. Treatments sharing the same lowercase letter within each building are not different at a significance level of 0.05.


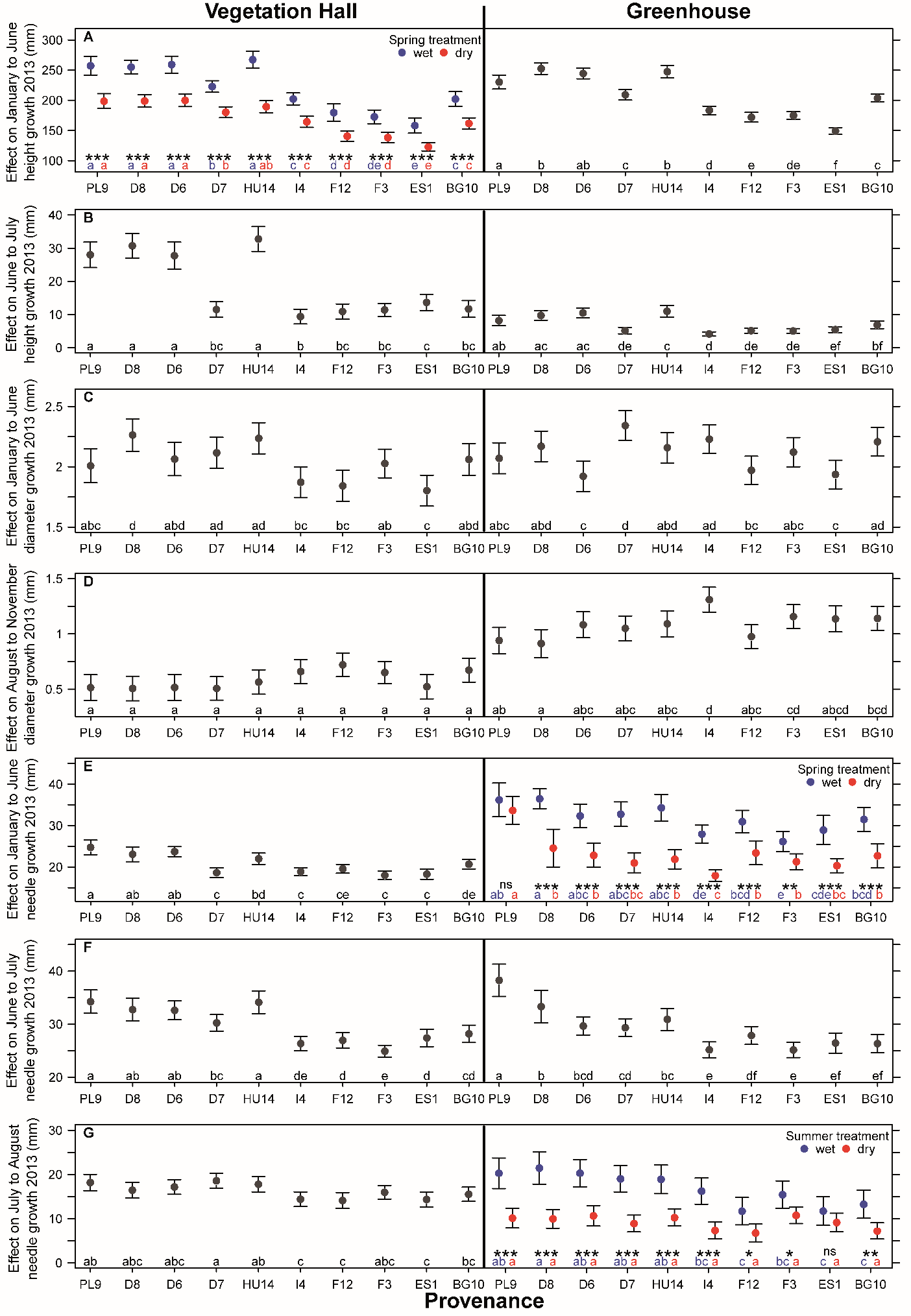
Supplementary Figure 8. Estimated model effects of provenance and provenance-drought-interactions on intra-annual (A, B) height growth, (C, D) diameter growth and (E – G) needle growth. Shown are fitted mean values and 95% confidence intervals holding all other variables included in the final model constant around their mean. Significance levels of asterisk are * *p* < 0.05, ** *p* < 0.01, *** *p* < 0.001, and ns *p* > 0.05. Provenances sharing the same lowercase letter in the same color within each building are not different at a significance level of 0.05. Provenance abbreviations are PL9 (Suprasl, Poland), D8 (Mittel-/Ostdt. Tiefland, Germany), D6 (Hauptsmoorwald, Germany), D7 (Alpenkiefer, Germany), HU14 (Plantage Pornoapati, Hungary), I4 (Emilia Romagna, Italy), F12 (Mont Ventoux, France), F3 (Prealpes du Sud, France), ES1 (Alto Ebro, Spain) and BG10 (Garmen, Bulgaria).


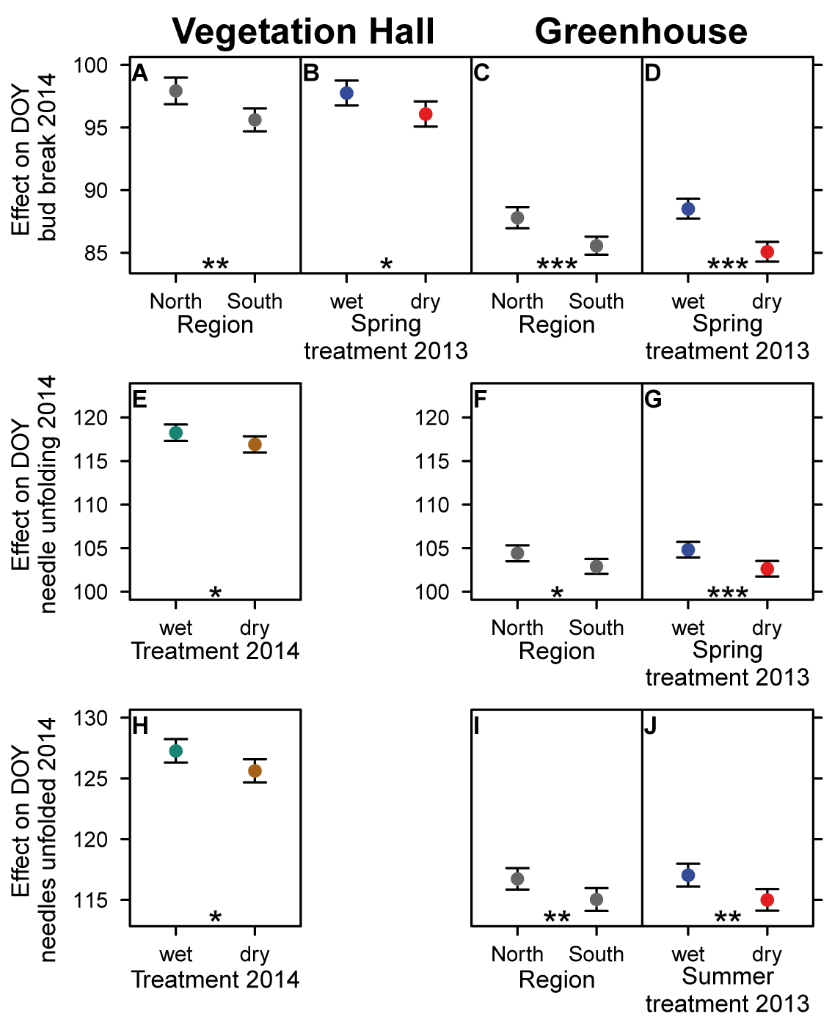


Supplementary Figure 9. Estimated model effects of region and drought treatments of 2013 and 2014 on the onset of (A – D) bud break, (E – G) needle unfolding and (H – J) the day when needles were unfolded in 2014. Shown are fitted mean values and 95% confidence intervals holding all other variables included in the final model constant around their mean. Significance levels of asterisk are * *p* < 0.05, ** *p* < 0.01, *** *p* < 0.001.


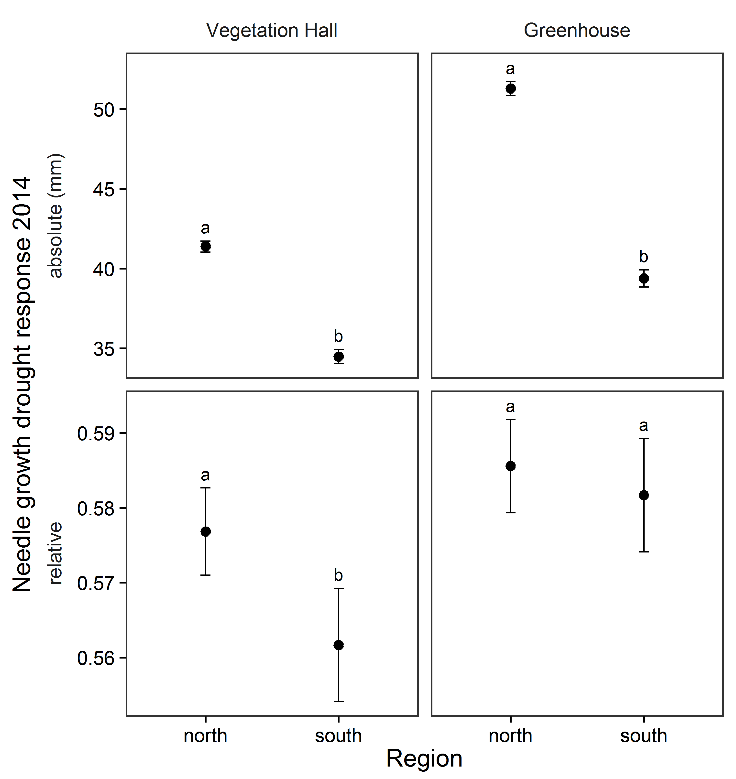


Supplementary Figure 10. Absolute and relative response to the spring drought in 2014 for needle growth from November 2013 to June 2014. Higher values indicate a stronger drought response. Differing letters within each panel indicate significant differences between region groups at a level of at least 0.05.


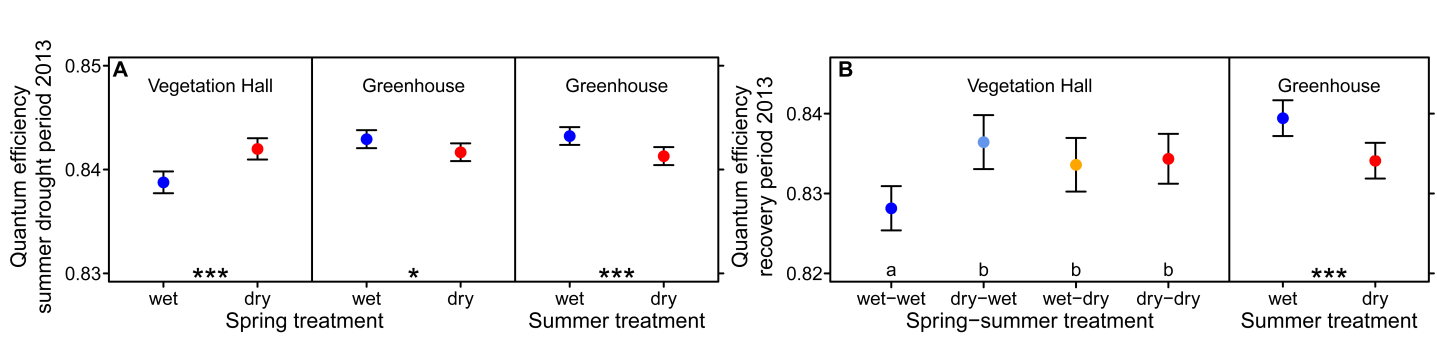
Supplementary Figure 11. Estimated model effects of the drought treatments of 2013 on the quantum efficiency of the photosystem II (A) during the summer drought 2013 and (B) during the recovery period three weeks after the end of the summer drought period 2013. Shown are fitted mean values and 95% confidence intervals. Significance levels of asterisk are * *p* < 0.05, ** *p* < 0.01, *** *p* < 0.001. Treatments sharing the same lowercase letter within each building are not different at a significance level of 0.05.


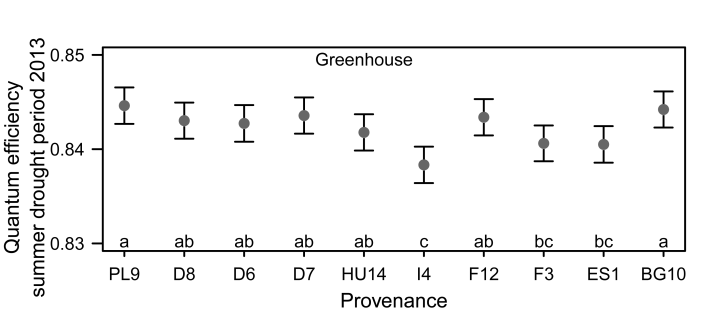


Supplementary Figure 12. Estimated model effect of provenance on the quantum efficiency of the Photosystem II in the greenhouse during the summer drought 2013 Shown are fitted mean values and 95% confidence intervals. Provenances sharing the same lowercase letter are not different at a significance level of 0.05. Provenance abbreviations are PL9 (Suprasl, Poland), D8 (Mittel-/Ostdt. Tiefland, Germany), D6 (Hauptsmoorwald, Germany), D7 (Alpenkiefer, Germany), HU14 (Plantage Pornoapati, Hungary), I4 (Emilia Romagna, Italy), F12 (Mont Ventoux, France), F3 (Prealpes du Sud, France), ES1 (Alto Ebro, Spain) and BG10 (Garmen, Bulgaria).


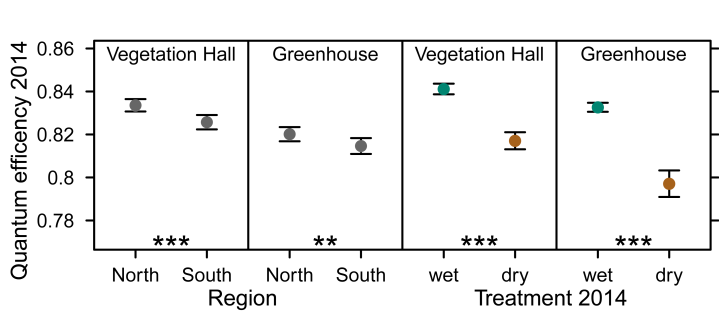


Supplementary Figure 13. Estimated model effects of the regional provenance group and the drought treatment on the quantum efficiency of the Photosystem II in 2014. Shown are fitted mean values and 95% confidence intervals. Significance levels of asterisk are ** *p* < 0.01, *** *p* < 0.001.


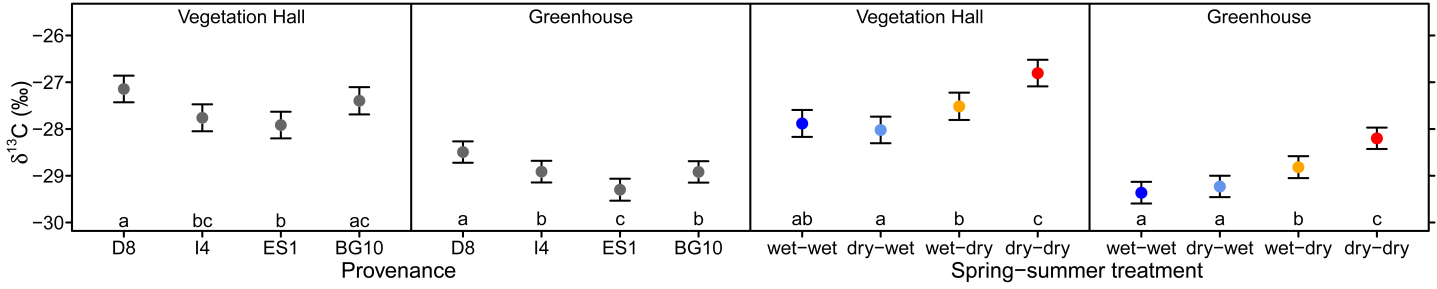
Supplementary Figure 14. Estimated model effects of provenance and the drought treatments of 2013 on the stable carbon isotope ratio of needles at the end of the summer drought period in 2013. Shown are fitted mean values and 95% confidence intervals. Provenances and treatments sharing the same lowercase letter within each building are not different at a significance level of 0.05. Provenance abbreviations are D8 (Mittel-/Ostdt. Tiefland, Germany), I4 (Emilia Romagna, Italy), ES1 (Alto Ebro, Spain) and BG10 (Garmen, Bulgaria).

## Supplementary Tables

Supplementary Table 1: Estimates (Est), respective Wald-values (Wald), p-values (p) and degrees of freedom (df) of variables remaining in the final models with the lowest AIC evaluating the effect of provenance and spring treatment on the phenophases bud burst (BBCH7), needles unfolding (BBCH9) and needles unfolded (BBCH16) in the vegetation hall (VegH) and greenhouse (GH) during 2013.

| Phenophase 2013 | BBCH7 | | | BBCH9 | | | BBCH16 | | |
| --- | --- | --- | --- | --- | --- | --- | --- | --- | --- |
| Building | VegH | | | VegH | | | VegH | | |
|  | Est | Wald | p | Est | Wald | p | Est | Wald | p |
| Spring treatment (df = 1) | ─ | ─ | ─ | 1.30 | 26.22 | <0.001 | 5.18 | 395.48 | <0.001 |
| Provenance (df = 9) | X | 39.61 | <0.001 | X | 97.37 | <0.001 | X | 55.51 | <0.001 |
| Phenophase 2013 | BBCH7 | | | BBCH9 | | | BBCH16 | | |
| Building | GH | | | GH | | | GH | | |
|  | Est | Wald | p | Est | Wald | p | Est | Wald | p |
| Spring treatment (df = 1) | ─ | ─ | ─ | ─ | ─ | ─ | 2.00 | 22.16 | <0.001 |
| Provenance (df = 9) | X | 70.30 | <0.001 | ─ | ─ | ─ | X | 70.08 | <0.001 |

Estimates of variables are extracted from the summary table of the respective models. "–" indicates that these variable was not included in all final models. Estimates show differences in comparison to the well-watered treatment. The effect sizes of single categories of provenance are not shown, but their contribution is indicated by X. "–" indicates that these variable was not included in all final models.

Supplementary Table 2: Estimates (Est), respective Wald-values (Wald), p-values (p) and degrees of freedom (df) of variables remaining in the final models with the lowest AIC evaluating the effect of provenance, spring treatment and covariates (DOY of bud burst 2013 (BBCH7), seedling height in January 2013) on height growth in the vegetation hall (VegH) and greenhouse (GH) during different periods of 2013.

| Height growth period 2013 | Jan-Jun | | | Jun-Jul | | | Jan-Jul | | |
| --- | --- | --- | --- | --- | --- | --- | --- | --- | --- |
| Building | VegH | | | VegH | | | VegH | | |
|  | Est | Wald | p | Est | Wald | p | Est | Wald | p |
| Spring treatment (df = 1) | -48.19 | 1.37 | 0.24 | ─ |  |  | -46.91 | 4.52 | 0.034 |
| Provenance (df = 9) | X | 336.18 | <0.001 | X | 305.61 | <0.001 | X | 365.24 | <0.001 |
| Height January (df = 1) | 0.17 | 10.75 | 0.001 | 0.045 | 14.96 | <0.001 | 0.13 | 3.92 | 0.048 |
| BBCH7 (df = 1) | -0.89 | 51.13 | <0.001 | ─ | ─ | ─ | -1.0 | 43.24 | <0.001 |
| Spring treatment:  Provenance (df = 9) | X | 26.17 | 0.002 | ─ | ─ | ─ | X | 20.50 | 0.015 |
| Spring treatment:  height January (df = 1) | -0.32 | 23.89 | <0.001 | ─ | ─ | ─ | -0.22 | 7.42 | 0.006 |
| Height growth period 2013 | Jan-Jun | | | Jun-Jul | | | Jan-Jul | | |
| Building | GH | | | GH | | | GH | | |
|  | Est | Wald | p | Est | Wald | p | Est | Wald | p |
| Spring treatment (df = 1) | -65.26 | 10.00 | 0.002 | 0.47 | 11.15 | <0.001 | -63.94 | 12.48 | <0.001 |
| Provenance (df = 9) | X | 774.92 | <0.001 | X | 144.75 | <0.001 | X | 857.13 | <0.001 |
| Height January (df = 1) | 0.20 | 11.44 | <0.001 | -0.019 | 12.55 | <0.001 | 0.17 | 8.62 | 0.003 |
| BBCH7 (df = 1) | -1.91 | 175.55 | <0.001 | ─ | ─ | ─ | -1.90 | 167.54 | <0.001 |
| Spring treatment:  height January (df = 1) | -0.29 | 15.20 | <0.001 | 0.032 | 17.41 | <0.001 | -0.24 | 10.16 | 0.001 |

Estimates of variables are extracted from the summary table of the respective models. Estimates show differences in comparison to the well-watered treatments. The effect sizes of single categories of provenance and its interactions are not shown, but their contribution is indicated by X. Estimated differences of categorical variables are calculated holding all continuous covariates constant around their respective mean. "–" indicates that these variable was not included in all final models.

Supplementary Table 3: Estimates (Est), respective Wald-values (Wald), p-values (p) and degrees of freedom (df) of variables remaining in the final models with the lowest AIC evaluating the effect of provenance, spring treatment, summer treatment and covariates (DOY of bud burst 2013 (BBCH7) and needles unfolding 2013 (BBCH9), seedling diameter in January 2013) on diameter growth in the vegetation hall (VegH) and greenhouse (GH) during different periods of 2013.

| Diameter growth period 2013 | Jan-Jun | | | Jun-Jul | | | Jul-Aug | | | Aug-Nov | | | Jan-Nov | | |
| --- | --- | --- | --- | --- | --- | --- | --- | --- | --- | --- | --- | --- | --- | --- | --- |
| Building | VegH | | | VegH | | | VegH | | | VegH | | | VegH | | |
|  | Est | Wald | p | Est | Wald | p | Est | Wald | p | Est | Wald | p | Est | Wald | p |
| Spring treatment (df = 1) | -0.47 | 8.28 | 0.004 | 0.040 | 13.53 | <0.001 | 0.0027 | 2.25 | 0.13 | -0.072 | 4.25 | 0.039 | -0.50 | 0.29 | 0.59 |
| Summer treatment (df = 1) | ─ | ─ | ─ | ─ | ─ | ─ | -0.97 | 1.95 | 0.16 | 0.22 | 38.81 | <0.001 | -0.83 | 2.02 | 0.15 |
| Provenance (df = 9) | X | 50.16 | <0.001 | ─ | ─ | ─ | ─ | ─ | ─ | X | 20.49 | 0.015 | X | 22.56 | 0.007 |
| Diameter January (df = 1) | 0.14 | 18.83 | <0.001 | 0.13 | 14.81 | <0.001 | 0.067 | 3.33 | 0.068 | 0.11 | 30.14 | <0.001 | 0.30 | 41.07 | <0.001 |
| BBCH7 (df = 1) | ─ | ─ | ─ | ─ | ─ | ─ | ─ | ─ | ─ | ─ | ─ | ─ | ─ | ─ | ─ |
| BBCH9 (df = 1) | -0.032 | 16.24 | <0.001 | 0.015 | 7.26 | 0.007 | ─ | ─ | ─ | -0.022 | 25.34 | <0.001 | -0.025 | 2.42 | 0.12 |
| Spring treatment:  summer treatment (df = 1) |  |  |  |  |  |  | -0.85 | 5.37 | 0.020 | ─ |  |  | -1.17 | 5.00 | 0.025 |
| Spring treatment:  diameter January (df = 1) | -0.18 | 16.75 | <0.001 | -0.16 | 12.70 | <0.001 | -0.078 | 2.33 | 0.13 | ─ |  |  | -0.29 | 21.05 | <0.001 |
| Summer treatment:  diameter January (df = 1) | ─ | ─ | ─ | ─ | ─ | ─ | -0.25 | 22.64 | <0.001 | ─ | ─ | ─ | ─ | ─ | ─ |
| Spring treatment:  summer treatment:  diameter January (df = 1) | ─ | ─ | ─ | ─ | ─ | ─ | -0.14 | 6.95 | 0.008 | ─ | ─ | ─ | ─ | ─ | ─ |
| Spring treatment:  BBCH9 (df = 1) | 0.033 | 10.08 | 0.001 | ─ | ─ | ─ | ─ | ─ | ─ | ─ | ─ | ─ | -0.0038 | 0.03 | 0.87 |
| Summer treatment:  BBCH9 (df = 1) | ─ | ─ | ─ | ─ | ─ | ─ | ─ | ─ | ─ | ─ | ─ | ─ | -0.037 | 2.94 | 0.086 |
| Spring treatment:  summer treatment:  BBCH9 (df = 1) | ─ | ─ | ─ | ─ | ─ | ─ | ─ | ─ | ─ | ─ | ─ | ─ | 0.027 | 5.20 | 0.023 |

Estimates of variables are extracted from the summary table of the respective models besides the two-way interactions of categorical variables (and its single compounds) and the three-way interactions of categorical variables with continuous variables which were calculated using the lsmean function and glht function, respectively. Estimates show differences in comparison to the well-watered treatments. The effect sizes of categorical variables with more than two levels (provenance) are not shown, but their contribution is indicated by X. Estimated differences of categorical variables are calculated holding all continuous covariates constant around their respective mean. "–" indicates that these variable was not included in all final models.

Supplementary Table 3 continued

| Diameter growth period 2013 | Jan-Jun | | | Jun-Jul | | | Jul-Aug | | | Aug-Nov | | | Jan-Nov | | |
| --- | --- | --- | --- | --- | --- | --- | --- | --- | --- | --- | --- | --- | --- | --- | --- |
| Building | GH | | | GH | | | GH | | | GH | | | GH | | |
|  | Est | Wald | p | Est | Wald | p | Est | Wald | p | Est | Wald | p | Est | Wald | p |
| Spring treatment (df = 1) | -0.49 | 1.08 | 0.30 | -0.12 | 2.84 | 0.092 | -0.27 | 69.86 | <0.001 | 0.32 | 75.45 | <0.001 | -0.52 | 0.34 | 0.56 |
| Summer treatment (df = 1) | ─ | ─ | ─ | ─ | ─ | ─ | -0.81 | 0.58 | 0.45 | 0.20 | 31.19 | <0.001 | -0.59 | 9.16 | 0.002 |
| Provenance (df = 9) | X | 42.71 | <0.001 | ─ | ─ | ─ | ─ | ─ | ─ | X | 35.02 | <0.001 | X | 35.64 | <0.001 |
| Diameter January (df = 1) | 0.087 | 10.04 | 0.002 | 0.043 | 3.55 | 0.059 | 0.0041 | 0.011 | 0.92 | 0.060 | 9.31 | 0.002 | 0.11 | 5.37 | 0.020 |
| BBCH7 (df = 1) | ─ | ─ | ─ | ─ | ─ | ─ | ─ | ─ | ─ | ─ | ─ | ─ | -0.017 | 5.63 | 0.018 |
| BBCH9 (df = 1) | ─ | ─ | ─ | ─ | ─ | ─ | -0.011 | 10.94 | 0.001 | ─ | ─ | ─ | ─ | ─ | ─ |
| Spring treatment:  summer treatment (df = 1) | ─ | ─ | ─ | ─ | ─ | ─ | ─ | ─ | ─ | ─ | ─ | ─ | -1.09 | 5.79 | 0.016 |
| Spring treatment:  diameter January (df = 1) | -0.13 | 11.79 | 0.001 | -0.081 | 5.77 | 0.016 | ─ | ─ | ─ | ─ | ─ | ─ | -0.15 | 5.90 | 0.015 |
| Summer treatment:  diameter January (df = 1) | ─ | ─ | ─ | ─ | ─ | ─ | -0.13 | 16.37 | <0.001 | ─ | ─ | ─ | ─ | ─ | ─ |
| Spring treatment:  BBCH7 (df = 1) | ─ | ─ | ─ | ─ | ─ | ─ | ─ | ─ | ─ | ─ | ─ | ─ | 0.0097 | 0.93 | 0.34 |
| Summer treatment:  BBCH7 (df = 1) | ─ | ─ | ─ | ─ | ─ | ─ | ─ | ─ | ─ | ─ | ─ | ─ | 0.024 | 5.43 | 0.020 |
| Spring treatment:  summer treatment:  BBCH7 (df = 1) | ─ | ─ | ─ | ─ | ─ | ─ | ─ | ─ | ─ | ─ | ─ | ─ | 0.00023 | 5.76 | 0.016 |

Supplementary Table 4: Estimates (Est), respective Wald-values (Wald), p-values (p) and degrees of freedom (df) of variables remaining in the final models with the lowest AIC evaluating the effect of provenance, spring treatment, summer treatment and the covariate DOY of needles unfolding 2013 (BBCH9) on needle growth in the vegetation hall (VegH) and greenhouse (GH) during different periods of 2013.

| Needle growth  period 2013 | Jan-Jun | | | Jun-Jul | | | Jul-Aug | | | Jan-Aug | | |
| --- | --- | --- | --- | --- | --- | --- | --- | --- | --- | --- | --- | --- |
| Building | VegH | | | VegH | | | VegH | | | VegH | | |
|  | Est | Wald | p | Est | Wald | p | Est | Wald | p | Est | Wald | p |
| Spring treatment (df = 1) | -6.33 | 15.16 | <0.001 | -0.64 | 5.88 | 0.015 | 10.39 | 93.81 | <0.001 | 3.61 | 7.70 | 0.006 |
| Summer treatment (df = 1) |  |  |  |  |  |  | -4.22 | 29.06 | <0.001 | -5.18 | 40.62 | <0.001 |
| Provenance (df = 9) | X | 114.77 | <0.001 | X | 146.35 | <0.001 | X | 32.63 | <0.001 | X | 134.19 | <0.001 |
| BBCH9 (df = 1) | -0.77 | 96.22 | <0.001 | -0.58 | 30.48 | <0.001 | -0.31 | 16.63 | <0.001 | -1.73 | 70.05 | <0.001 |
| Spring treatment:  summer treatment (df = 1) | ─ | ─ | ─ | ─ | ─ | ─ | 1.01 | 16.69 |  | -6.55 | 4.56 | 0.033 |
| Spring treatment:  BBCH9 (df = 1) | 0.34 | 11.67 | 0.001 | 0.32 | 5.74 | 0.017 | ─ | ─ | ─ | 0.84 | 7.86 | 0.005 |
| Needle growth  period 2013 | Jan-Jun | | | Jun-Jul | | | Jul-Aug | | | Jan-Aug | | |
| Building | GH | | | GH | | | GH | | | GH | | |
|  | Est | Wald | p | Est | Wald | p | Est | Wald | p | Est | Wald | p |
| Spring treatment (df = 1) | -8.79 | 0.89 | 0.35 | -0.22 | 5.01 | 0.025 | 11.23 | 119.62 | <0.001 | 1.76 | 1.25 | 0.26 |
| Summer treatment (df = 1) |  |  |  |  |  |  | -3.91 | 8.61 | 0.003 | -0.77 | 0.20 | 0.66 |
| Provenance (df = 9) | X | 56.46 | <0.001 | X | 103.99 | <0.001 | X | 44.27 | <0.001 | X | 162.07 | <0.001 |
| BBCH9 (df = 1) | -0.44 | 46.01 | <0.001 | -0.36 | 13.08 | <0.001 | ─ | ─ | ─ | -0.6 | 29.15 | <0.001 |
| Spring treatment:  summer treatment (df = 1) | ─ | ─ | ─ | ─ | ─ | ─ | -0.24 | 38.79 | <0.001 | -7.70 | 14.18 | <0.001 |
| Spring treatment:  provenance (df = 9) | X | 20.01 | 0.018 | ─ | ─ | ─ | ─ | ─ | ─ | ─ | ─ | ─ |
| Summer treatment:  provenance (df = 9) | ─ | ─ | ─ | ─ | ─ | ─ | X | 20.30 | 0.016 | ─ | ─ | ─ |
| Spring treatment:  BBCH9 (df = 1) | ─ | ─ | ─ | 0.29 | 4.96 | 0.026 | ─ | ─ | ─ | ─ | ─ | ─ |

Estimates of variables are extracted from the summary table of the respective models besides the two-way interactions of categorical variables (and its single compounds) which were calculated using the lsmean function. Estimates show differences in comparison to the well-watered treatments. The effect sizes of categorical variables with more than two levels (provenance) are not shown, but their contribution is indicated by X. Estimated differences of categorical variables are calculated holding all continuous covariates constant around their respective mean. "–" indicates that these variable was not included in all final models.

Supplementary Table 5: Estimates (Est), respective Wald-values (Wald), p-values (p) and degrees of freedom (df) of variables remaining in the final models with the lowest AIC evaluating the effect of provenance, spring treatment, summer treatment and covariates (DOY of bud burst 2013 (BBCH7) and needles unfolding 2013 (BBCH9)) on the biomass of different compartments and total biomass in the vegetation hall (VegH) and greenhouse (GH) in 2013.

| Biomass of compartments 2013 | wood | | | needle | | | bud | | | total | | | needle/wood | | |
| --- | --- | --- | --- | --- | --- | --- | --- | --- | --- | --- | --- | --- | --- | --- | --- |
| Building | VegH | | | VegH | | | VegH | | | VegH | | | VegH | | |
|  | Est | Wald | p | Est | Wald | p | Est | Wald | p | Est | Wald | p | Est | Wald | p |
| Spring treatment (df = 1) | -1.60 | 36.33 | <0.001 | -0.17 | 1.58 | 0.21 | -0.45 | 17.55 | <0.001 | -2.14 | 14.10 | <0.001 | 0.17 | 34.15 | <0.001 |
| Summer treatment (df = 1) | -1.77 | 4.87 | 0.027 | -1.49 | 0.88 | 0.35 | -0.10 | 85.08 | <0.001 | -3.68 | 42.56 | <0.001 | 0.10 | 5.24 | 0.022 |
| Provenance (df = 9) | X | 66.17 | <0.001 | X | 59.25 | 0.024 | X | 34.98 | <0.001 | X | 59.92 | <0.001 | X | 63.01 | <0.001 |
| BBCH7 (df = 1) | ─ | ─ | ─ | ─ | ─ | ─ | ─ | ─ | ─ | ─ | ─ | ─ | ─ | ─ | ─ |
| BBCH9 (df = 1) | -0.024 | 0.25 | 0.62 | 0.030 | 0.13 | 0.72 | ─ |  |  | ─ |  |  | 0.0021 | 0.37 | 0.54 |
| Spring treatment:summer treatment (df = 1) | ─ | ─ | ─ | -2.37 | 5.10 | 0.024 | ─ | ─ | ─ | ─ | ─ | ─ | 0.17 | 5.05 | 0.025 |
| Spring treatment:BBCH9 (df = 1) | ─ | ─ | ─ | -0.14 | 1.62 | 0.20 | ─ | ─ | ─ | ─ | ─ | ─ | ─ | ─ | ─ |
| Summer treatment:  BBCH9 (df = 1) | 0.12 | 3.92 | 0.048 | -0.12 | 1.06 | 0.30 | ─ | ─ | ─ | ─ | ─ | ─ | -0.010 | 4.49 | 0.034 |
| Spring treatment:  summer treatment:  BBCH9 (df = 1) | ─ | ─ | ─ | 0.083 | 4.93 | 0.026 | ─ | ─ | ─ | ─ | ─ | ─ | ─ | ─ | ─ |

Estimates of variables are extracted from the summary table of the respective models besides the two-way interactions of categorical variables (and its single compounds) and the three-way interactions of categorical variables with continuous variables which were calculated using the lsmean function and glht function, respectively. Estimates show differences in comparison to the well-watered treatments. The effect sizes of categorical variables with more than two levels (provenance) are not shown, but their contribution is indicated by X. Estimated differences of categorical variables are calculated holding all continuous covariates constant around their respective mean. "–" indicates that these variable was not included in all final models.

Supplementary Table 5 continued

| Biomass of compartments 2013 | wood | | | needle | | | bud | | | total | | | needle/wood | | |
| --- | --- | --- | --- | --- | --- | --- | --- | --- | --- | --- | --- | --- | --- | --- | --- |
| Building | GH | | | GH | | | GH | | | GH | | | GH | | |
|  | Est | Wald | p | Est | Wald | p | Est | Wald | p | Est | Wald | p | Est | Wald | p |
| Spring treatment (df = 1) | -2.66 | 108.78 | <0.001 | -0.49 | 1.32 | 0.25 | -0.071 | 68.18 | <0.001 | -3.67 | 56.19 | <0.001 | 0.27 | 7.46 | 0.006 |
| Summer treatment (df = 1) | -2.11 | 81.24 | <0.001 | -1.02 | 6.78 | 0.009 | -0.099 | 131.38 | <0.001 | -3.99 | 68.20 | <0.001 | 0.19 | 34.31 | <0.001 |
| Provenance (df = 9) | X | 94.40 | <0.001 | X | 72.92 | <0.001 | X | 69.59 | <0.001 | X | 80.37 | <0.001 | X | 43.80 | <0.001 |
| BBCH7 (df = 1) | ─ | ─ | ─ | ─ | ─ | ─ | ─ | ─ | ─ | ─ | ─ | ─ | 0.0037 | 9.71 | 0.002 |
| BBCH9 (df = 1) | ─ | ─ | ─ | ─ | ─ | ─ | -0.0028 | 8.36 | 0.004 | ─ | ─ | ─ | 0.0037 | 1.37 | 0.24 |
| Spring treatment:summer treatment | ─ | ─ | ─ | -2.83 | 5.94 | 0.015 | ─ | ─ | ─ | ─ | ─ | ─ | 0.31 | 15.75 | <0.001 |
| Summer treatment:  provenance (df = 9) | ─ | ─ | ─ | ─ | ─ | ─ | ─ | ─ | ─ | ─ | ─ | ─ | X | 17.62 | 0.040 |
| Spring treatment:BBCH9 (df = 1) | ─ | ─ | ─ | ─ | ─ | ─ | ─ | ─ | ─ | ─ | ─ | ─ | -0.0087 | 4.82 | 0.028 |

Supplementary Table 6: Estimates (Est), respective Wald-values (Wald), p-values (p) and degrees of freedom (df) of variables remaining in the final models with the lowest AIC evaluating the effect of provenance region, spring treatment 2013, summer treatment 2013 and treatment 2014 on the phenophases bud burst (BBCH7), needles unfolding (BBCH9) and needles unfolded (BBCH16) in the vegetation hall (VegH) and greenhouse (GH) during 2014.

| Phenophase 2014 | BBCH7 | | | BBCH9 | | | BBCH16 | | |
| --- | --- | --- | --- | --- | --- | --- | --- | --- | --- |
| Building | VegH | | | VegH | | | VegH | | |
|  | Est | Wald | p | Est | Wald | p | Est | Wald | p |
| Spring treatment 2013 (df = 1) | -1.68 | 5.67 | 0.017 | ─ | ─ | ─ | ─ | ─ | ─ |
| Treatment 2014 (df = 1) | ─ | ─ | ─ | -1.33 | 3.90 | 0.048 | -1.64 | 5.71 | 0.017 |
| Region (df = 1) | -2.31 | 10.57 | 0.001 | ─ | ─ | ─ | ─ | ─ | ─ |
| Phenophase 2014 | BBCH7 | | | BBCH9 | | | BBCH16 | | |
| Building | GH | | | GH | | | GH | | |
|  | Est | Wald | p | Est | Wald | p | Est | Wald | p |
| Spring treatment 2013 (df = 1) | -3.38 | 35.31 | <0.001 | -2.18 | 11.91 | 0.001 | ─ | ─ | ─ |
| Summer treatment 2013 (df = 1) | ─ | ─ | ─ | ─ | ─ | ─ | -2.03 | 9.66 | 0.002 |
| Region (df = 1) | -2.23 | 15.23 | <0.001 | -1.51 | 5.67 | 0.017 | -1.70 | 6.77 | 0.009 |

Estimates of variables are extracted from the summary table of the respective models besides for two-way interactions of categorical variables and its single compounds which were calculated using the lsmean function. Estimates show differences in comparison to the well-watered treatments or/and the reference region north. "–" indicates that these variable was not included in all final models.

Supplementary Table 7: Estimates (Est), respective Wald-values (Wald), p-values (p) and degrees of freedom (df) of variables remaining in the final models with the lowest AIC evaluating the effect of provenance region, spring treatment 2013, summer treatment 2013, treatment 2014 and covariates (DOY of bud burst 2014 (BBCH7), seedling height in January 2013) on height growth in the vegetation hall (VegH) and greenhouse (GH) during 2014.

| Height growth period 2014 | Nov-Jun | | | Nov-Jun | | |
| --- | --- | --- | --- | --- | --- | --- |
| Building | VegH | | | GH | | |
|  | Est | Wald | p | Est | Wald | p |
| Spring treatment 2013 (df = 1) | 13.46 | 4.80 | 0.029 | 17.12 | 4.15 | 0.042 |
| Summer treatment 2013 (df = 1) | -34.99 | 11.87 | 0.001 | ─ | ─ | ─ |
| Treatment 2014 (df = 1) | -76.28 | 73.47 | <0.001 | -32.61 | 17.17 | <0.001 |
| Region (df = 1) | -25.7 | 16.76 | <0.001 | -27.15 | 10.90 | 0.001 |
| BBCH7 (df = 1) | -2.87 | 32.11 | <0.001 | -1.82 | 4.24 | 0.039 |
| Summer treatment 2013:treatment 2014 (df = 1) | -75.26 | 8.12 | 0.004 | ─ | ─ | ─ |

Estimates of variables are extracted from the summary table of the respective models besides for two-way interactions of categorical variables and its single compounds which were calculated using the lsmean function. Estimates show differences in comparison to the well-watered treatments or the reference region north. Estimated differences of categorical variables are calculated holding all continuous covariates constant around their respective mean "–" indicates that these variable was not included in all final models.

Supplementary Table 8: Estimates (Est), respective Wald-values (Wald), p-values (p) and degrees of freedom (df) of variables remaining in the final models with the lowest AIC evaluating the effect of provenance region, spring treatment 2013, summer treatment 2013, treatment 2014 and covariates (DOY of bud burst 2014 (BBCH7) and needles unfolding 2014 (BBCH9)) on diameter growth in the vegetation hall (VegH) and greenhouse (GH) during 2014.

| Diameter growth period 2014 | Nov-Jun | | | Nov-Jun | | |
| --- | --- | --- | --- | --- | --- | --- |
| Building | VegH | | | GH | | |
|  | Est | Wald | p | Est | Wald | p |
| Spring treatment 2013 (df = 1) | 0.78 | 34.19 | <0.001 | ─ | ─ | ─ |
| Summer treatment 2013 (df = 1) | 0.62 | 21.92 | <0.001 | ─ | ─ | ─ |
| Treatment 2014 (df = 1) | -2.26 | 38.86 | <0.001 | -2.39 | 16.97 | <0.001 |
| Region (df = 1) | 0.29 | 8.16 | 0.004 | 0.21 | 3.63 | 0.057 |
| BBCH7 | ─ | ─ | ─ | -0.03 | 5.53 | 0.019 |
| BBCH9 (df = 1) | -0.092 | 42.60 | <0.001 | -0.047 | 5.54 | 0.019 |
| Spring treatment 2013:  summer treatment 2013 (df = 1) | 0.82 | 9.47 | 0.002 | ─ | ─ | ─ |
| Treatment 2014:BBCH9 (df = 1) | 0.088 | 26.46 | <0.001 | 0.068 | 9.50 | 0.002 |

Estimates of variables are extracted from the summary table of the respective models besides for two-way interactions of categorical variables and its single compounds which were calculated using the lsmean function. Estimates show differences in comparison to the well-watered treatments or the reference region north. Estimated differences of categorical variables are calculated holding all continuous covariates constant around their respective mean "–" indicates that these variable was not included in all final models.

Supplementary Table 9: Estimates (Est), respective Wald-values (Wald), p-values (p) and degrees of freedom (df) of variables remaining in the final models with the lowest AIC evaluating the effect of provenance region, spring treatment 2013, summer treatment 2013, treatment 2014 and DOY of needles unfolding 2014 (BBCH9) on needle growth in the vegetation hall (VegH) and greenhouse (GH) during 2014.

| Needle growth 2014 | Jan-Jun | | | Jan-Jun | | |
| --- | --- | --- | --- | --- | --- | --- |
| Building | VegH | | | GH | | |
|  | Est | Wald | p | Est | Wald | p |
| Spring treatment 2013 (df = 1) | 3.02 | 10.75 | 0.001 | 3.78 | 12.17 | <0.001 |
| Summer treatment 2013 (df = 1) | 2.67 | 8.46 | 0.004 | 2.47 | 5.40 | 0.020 |
| Treatment 2014 (df = 1) | -42.78 | 33.05 | <0.001 | -51.69 | 25.86 | <0.001 |
| Region (df = 1) | -11.82 | 52.47 | <0.001 | -21.01 | 109.20 | <0.001 |
| BBCH9 (df = 1) | -1.05 | 48.93 | <0.001 | -0.93 | 18.37 | <0.001 |
| Treatment 2014:region (df = 1) | -46.58 | 16.38 | <0.001 | -60.15 | 27.48 | <0.001 |
| Treatment 2014:BBCH9 (df = 1) | 0.67 | 14.17 | <0.001 | 0.75 | 9.50 | 0.002 |

Estimates of variables are extracted from the summary table of the respective models besides for two-way interactions of categorical variables and its single compounds which were calculated using the lsmean function. Estimates show differences in comparison to the well-watered treatments or/and the reference region north. Estimated differences of categorical variables are calculated holding all continuous covariates constant around their respective mean. "–" indicates that these variable was not included in all final models.

Supplementary Table 10: Estimates (Est), respective Wald-values (Wald), p-values (p) and degrees of freedom (df) of variables remaining in the final models with the lowest AIC evaluating the effect of provenance, spring treatment and summer treatment on the quantum yield of chlorophyll fluorescence in the vegetation hall (VegH) and greenhouse (GH) during 2013.

| Quantum yield measurement phase 2013 | summer | | | recovery | | |
| --- | --- | --- | --- | --- | --- | --- |
| Building | VegH | | | VegH | | |
|  | Est | Wald | p | Est | Wald | p |
| Spring treatment (df = 1) | 0.0032 | 18.56 | <0.001 | 0.0083 | 13.89 | <0.001 |
| Summer treatment (df = 1) | ─ | ─ | ─ | 0.0054 | 6.05 | 0.014 |
| Spring treatment:summer treatment (df = 1) | ─ | ─ | ─ | 0.0061 | 5.47 | 0.019 |
| Quantum yield measurement phase 2013 | summer | | | recovery | | |
| Building | GH | | | GH | | |
|  | Est | Wald | p | Est | Wald | p |
| Spring treatment (df = 1) | -0.0013 | 4.13 | 0.042 | ─ | ─ | ─ |
| Summer treatment (df = 1) | -0.0019 | 9.70 | 0.002 | -0.0053 | 10.97 | 0.001 |
| Provenance (df = 9) | X | 35.79 | <0.001 | ─ | ─ | ─ |

Estimates of variables are extracted from the summary table of the respective models besides the two-way interactions of categorical variables (and its single compounds) which were calculated using the lsmean function. Estimates show differences in comparison to the well-watered treatments. "–" indicates that these variable was not included in all final models.

Supplementary Table 11: Estimates (Est), respective Wald-values (Wald), p-values (p) and degrees of freedom (df) of variables remaining in the final models with the lowest AIC evaluating the effect of provenance, spring treatment 2013, summer treatment 2013 and treatment 2014 on the quantum yield of chlorophyll fluorescence in the vegetation hall (VegH) and greenhouse (GH) during 2014.

| Quantum yield Spring 2014 |  |  |  |  |  |  |
| --- | --- | --- | --- | --- | --- | --- |
| Building | VegH | | | GH | | |
|  | Est | Wald | p | Est | Wald | p |
| Treatment 2014 (df = 1) | -0.024 | 102.61 | <0.001 | -0.036 | 115.66 | <0.001 |
| Region (df = 1) | -0.0079 | 13.25 | <0.001 | -0.0055 | 7.29 | 0.007 |

Estimates of variables are extracted from the summary table of the respective models. Estimates show differences in comparison to the well-watered treatment or the reference region north. "–" indicates that these variable was not included in all final models.

Supplementary Table 12: Estimates (Est), respective Wald-values (Wald), p-values (p) and degrees of freedom (df) of variables remaining in the final models with the lowest AIC evaluating the effect of provenance, spring treatment and summer treatment on stable carbon isotopes ratio in the vegetation hall (VegH) and greenhouse (GH).

| Stable carbon isotopes ratio 2013 |  |  |  |  |  |  |
| --- | --- | --- | --- | --- | --- | --- |
| Building | VegH | | | GH | | |
|  | Est | Wald | p | Est | Wald | p |
| Spring treatment (df = 1) | -0.14 | 0.46 | 0.50 | 0.13 | 0.66 | 0.42 |
| Summer treatment (df = 1) | 0.37 | 3.12 | 0.077 | 0.55 | 10.70 | 0.001 |
| Provenance (df = 9) | X | 17.56 | 0.001 | X | 23.61 | <0.001 |
| Spring treatment:  summer treatment (df = 1) | 1.08 | 8.53 | 0.003 | 1.16 | 4.28 | 0.039 |

The effect sizes and levels of significance (* *p* < 0.05, ** *p* < 0.01, *** *p* < 0.001, ns *p* > 0.05) are extracted from the summary table of the respective models besides the two-way interactions of categorical variables (and its single compounds) which were calculated using the lsmean function. Estimates show differences in comparison to the well-watered treatments. "–" indicates that these variable was not included in all final models.

Supplementary Table 13: Estimates (Est), respective Wald-values (Wald), p-values (p) and degrees of freedom (df) of variables remaining in the final models with the lowest AIC evaluating the effect of provenance, spring treatment and summer treatment and stable carbon isotope ratio (δ^13^C) on annual growth and biomass of 2013 in the vegetation hall (VegH).

| Response variable | height growth | | | diameter growth | | | needle growth | | |  |  |  |
| --- | --- | --- | --- | --- | --- | --- | --- | --- | --- | --- | --- | --- |
| Building | VegH | | | VegH | | | VegH | | |  |  |  |
|  | Est | Wald | p | Est | Wald | p | Est | Wald | p |  |  |  |
| Spring treatment (df = 1) | -40.14 | 27.08 | <0.001 | ─ | ─ | ─ | ─ | ─ | ─ |  |  |  |
| Summer treatment (df = 1) | ─ | ─ | ─ | -1.47─ | 75.98 | <0.001 | -9.17 | 6.72 | 0.010 |  |  |  |
| Provenance (df = 3) | X | 80.57 | <0.001 | ─ | ─ | ─ | X | 13.42 | 0.004 |  |  |  |
| δ^13^C (df = 1) | ─ | ─ | ─ | 0.42 | 28.98 | <0.001 | ─ | ─ | ─ |  |  |  |
| Response variable | total biomass  VegH | | | wood biomass | | | needle biomass | | | bud biomass | | |
| Building |  |  |  | VegH | | | VegH | | | VegH | | |
|  | Est | Wald | p | Est | Wald | p | Est | Wald | p | Est | Wald | p |
| Spring treatment (df = 1) | -4.12 | 13.98 | <0.001 | -1.58 | 5.98 | 0.015 | -2.02 | 9.15 | 0.002 | ─ | ─ | ─ |
| Summer treatment (df = 1) | -7.64 | 37.31 | <0.001 | -3.45 | 23.04 | <0.001 | -4.01 | 28.31 | <0.001 | -0.18 | 48.89 | <0.001 |
| Provenance (df = 3) | ─ | ─ | ─ | ─ | ─ | ─ | ─ | ─ | ─ | X | 4.64 | 0.02 |
| δ^13^C (df = 1) | 3.82 | 49.97 | <0.001 | 1.4 | 18.67 | <0.001 | 2.32 | 42.45 | <0.001 | 0.08 | 57.18 | <0.001 |

The effect sizes and levels of significance (* *p* < 0.05, ** *p* < 0.01, *** *p* < 0.001, ns *p* > 0.05) are extracted from the summary table of the respective models. Estimates show differences in comparison to the well-watered treatments. The effect sizes of categorical variables with more than two levels (provenance) are not shown, but their contribution is indicated by X. Estimated differences of categorical variables are calculated holding δ^13^C constant around its mean. "–" indicates that these variable was not included in all final models.

Supplementary Table 14: Estimates (Est), respective Wald-values (Wald), p-values (p) and degrees of freedom (df) of variables remaining in the final models with the lowest AIC evaluating the effect of provenance, spring treatment and summer treatment and stable carbon isotope ratio (δ^13^C) on annual growth and biomass of 2013 in the greenhouse (GH).

| Response variable | height growth | | | diameter growth | | | needle growth | | |  |  |  |
| --- | --- | --- | --- | --- | --- | --- | --- | --- | --- | --- | --- | --- |
| Building | GH | | | GH | | | GH | | |  |  |  |
|  | Est | Wald | p | Est | Wald | p | Est | Wald | p |  |  |  |
| Spring treatment (df = 1) | -77.45 | 113.25 | <0.001 | -0.46 | 11.31 | 0.001 | ─ | ─ | ─ |  |  |  |
| Summer treatment (df = 1) | ─ | ─ | ─ | -0.80 | 26.37 | <0.001 | -6.70 | 4.75 |  |  |  |  |
| Provenance (df = 3) | X | 10.24 | 0.017 | ─ | ─ | ─ | X | 27.83 |  |  |  |  |
| δ^13^C (df = 1) | -24.25 | 0.62 | 0.43 | 0.39 | 19.28 | <0.001 | ─ | ─ | ─ |  |  |  |
| δ^13^C : provenance (df = 3) | X | 12.18 | 0.007 | ─ | ─ | ─ | ─ | ─ | ─ |  |  |  |
| Response variable | total biomass | | | wood biomass | | | needle biomass | | | bud biomass | | |
| Building | GH | | | GH | | | GH | | | GH | | |
|  | Est | Wald | p | Est | Wald | p | Est | Wald | p | Est | Wald | p |
| Spring treatment (df = 1) | -2.22 | 20.58 | <0.001 | -4.32 | 47.71 | <0.001 | ─ | ─ | ─ | -0.19 | 23.16 | <0.001 |
| Summer treatment (df = 1) | -2.51 | 26.11 | <0.001 | -3.54 | 24.98 | <0.001 | -2.73 | 15.52 | <0.001 | -0.13 | 35.68 | <0.001 |
| Provenance (df = 3) | X | 12.92 | 0.005 | X | 15.46 | 0.001 | ─ | ─ | ─ | X | 13.59 | 0.004 |
| δ^13^C (df = 1) | 5.22 | 65.81 | <0.001 | 2.92 | 50.78 | <0.001 | 2.23 | 35.46 | <0.001 | 0.07 | 29.28 | <0.001 |
| Spring treatment:  summer treatment (df = 1) | -10.91 | 9.93 | 0.002 | ─ | ─ | ─ | ─ | ─ | ─ | ─ | ─ | ─ |

The effect sizes and levels of significance (* *p* < 0.05, ** *p* < 0.01, *** *p* < 0.001, ns *p* > 0.05) are extracted from the summary table of the respective models besides the two-way interactions of categorical variables (and its single compounds) which were calculated using the lsmean function. Estimates show differences comparison to the well-watered treatments. The effect sizes of categorical variables with more than two levels (provenance) are not shown, but their contribution is indicated by X. Estimated differences of categorical variables are calculated holding δ^13^C constant around its mean. "–" indicates that these variable was not included in all final models.

## References

Harris, I, P.D. Jones, T.J. Osborn, and D.H. Lister. 2014. “Updated High-Resolution Grids of Monthly Climatic Observations - the CRU TS3.10 Dataset.” *International Journal of Climatology* 34 (3): 623–42. doi:10.1002/joc.3711.
